# Individual structural features constrain the mouse functional connectome

**DOI:** 10.1073/pnas.1906694116

**Published:** 2019-12-11

**Authors:** Francesca Melozzi, Eyal Bergmann, Julie A. Harris, Itamar Kahn, Viktor Jirsa, Christophe Bernard

**Affiliations:** ^a^Aix Marseille Univ, Inserm, INS, Institut de Neurosciences des Systèmes, Marseille, France;; ^b^Department of Neuroscience, Rappaport Faculty of Medicine, Technion–Israel Institute of Technology, Haifa, 31096, Israel;; ^c^Allen Institute for Brain Science, Seattle, WA 98109

**Keywords:** connectome, MRI, resting state, large-scale model

## Abstract

Whole brain dynamics intuitively depend upon the internal wiring of the brain; but to which extent the individual structural connectome constrains the corresponding functional connectome is unknown, even though its importance is uncontested. After acquiring structural data from individual mice, we virtualized their brain networks and simulated in silico functional MRI data. Theoretical results were validated against empirical awake functional MRI data obtained from the same mice. We demonstrate that individual structural connectomes predict the functional organization of individual brains. Using a virtual mouse brain derived from the Allen Mouse Brain Connectivity Atlas, we further show that the dominant predictors of individual structure–function relations are the asymmetry and the weights of the structural links. Model predictions were validated experimentally using tracer injections, identifying which missing connections (not measurable with diffusion MRI) are important for whole brain dynamics in the mouse. Individual variations thus define a specific structural fingerprint with direct impact upon the functional organization of individual brains, a key feature for personalized medicine.

## Significance

The structural connectome is a key determinant of brain function and dysfunction. The connectome-based model approach aims to understand the functional organization of the brain by modeling the brain as a dynamical system, then studying how the functional architecture rises from the underlying structural skeleton. Here, taking advantage of mice studies, we systematically investigated the informative content of different structural features in explaining the emergence of the functional ones. We demonstrate that individual variations define a specific structural fingerprint with a direct impact upon the functional organization of individual brains stressing the importance of using individualized models to understand brain function. We show how limitations of connectome reconstruction with the diffusion-MRI method restrict our comprehension of the structural–functional relation.

Structural connectivity (SC) refers to a set of physical links between brain areas (connectome, ref. 1) and constitutes an individual fingerprint in humans (2, 3). Since the connectome provides the physical substrate for information flow in the brain, it should impose strong constraints on whole brain dynamics. Functional connectivity (FC), in the context of resting-state functional MRI, refers to coherent slow spontaneous fluctuations in the blood oxygenation level-dependent (BOLD) signals measured in the passive awake individual. FC is commonly used to assess whole brain dynamics and function (4). Similar to SC, FC constitutes an individual functional fingerprint (5–7) and shows specific alterations during aging and in brain disorders (8). There is thus a strong correlation between the structural and the functional connectome. However, the causal relation between SC and FC remains unknown. Large-scale brain modeling offers a way to explore causality between structural and functional connectivity. Combining experimental and theoretical approaches, we here unravel and quantify the degree to which the individual’s SC explains the same individual’s variations in FC.

We use The Virtual Brain (TVB), which allows building individual brain network models based on structural data (9). This brain network modeling approach operationalizes the functional consequences of structural network variations (10, 11) and allows us to systematically investigate SC–FC relations in individual human brains (12–15). If SC constrains FC, SC-based simulations of FC should match empirical FC within the bounds of validity of the metric. In primates and rodents, individual SCs are derived from diffusion MRI (dMRI). However, dMRI does not provide information on fiber directionality or synaptic details (distribution and type of neurotransmitter) and suffers from limitations, such as underestimation of fiber length and misidentification of crossing fiber tracks (16, 17). Given the imprecision of dMRI-derived SC, it is difficult to estimate the validity of the simulations. This would require the knowledge of the ground truth connectome of an individual, which cannot be measured at present. However, the currently best gold standard can be derived in mice from cellular-level tracing of axonal projections (18), here named the Allen connectome. Although individuality is lost (the SC is a composite of many mice) and despite other limitations (19, 20), the Allen connectome provides details not available otherwise and in particular not available in humans. Focusing our attention on simulating mouse brain dynamics, we can thus use this detailed connectome to explore which missing features in the dMRI account for individual SC–FC relations. Specifically, we predict that fiber directionality and fine grain connectivity patterns should be key determinants.

Using dMRI data of 19 mice, we constructed 19 virtual mouse brain models (21) and compared predicted FC with empirical FC data acquired from the same mice during passive wakefulness (22). We found that individual SC predicts individual FC better than the dMRI-based averaged SC, and that predictions can be improved by considering fiber directionality, coupling weights and specific fiber tracks derived from the Allen connectome. We also found that hemispherical lateralization in the mouse connectome influences whole brain dynamics.

## Results

**Fig. 1.**
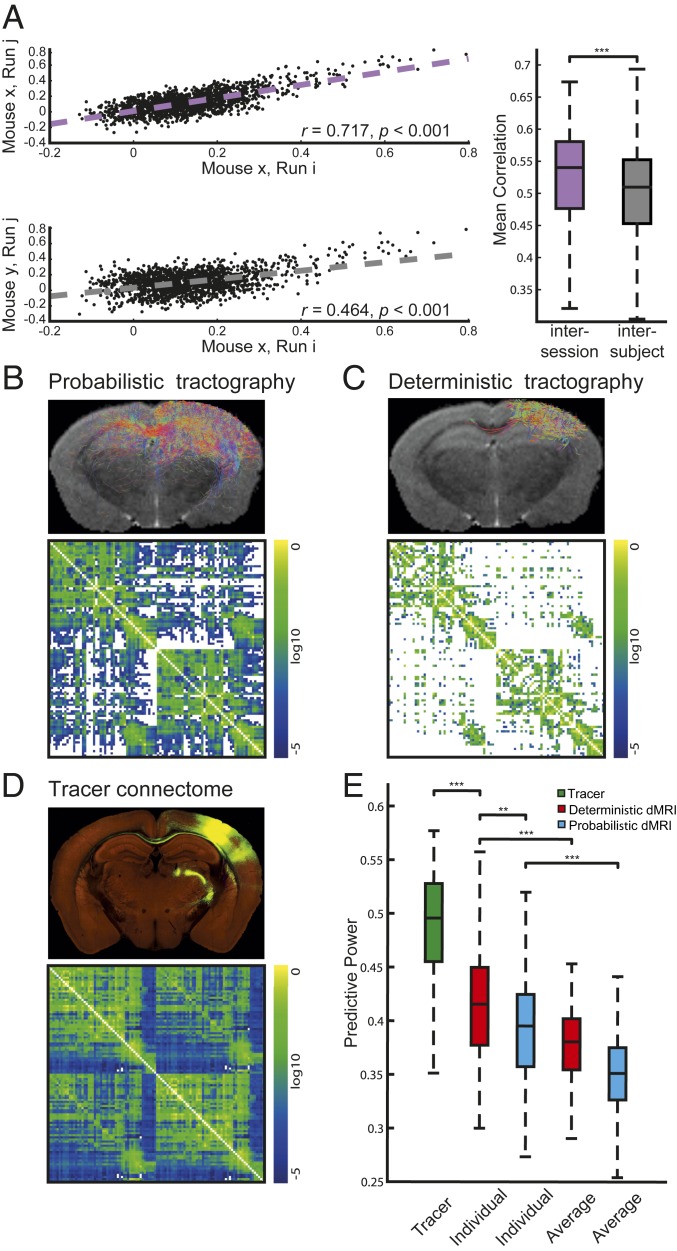
FC reliability was evaluated by comparing functional connectivity estimates between sessions. Representative scatterplots (*Left*) show the correlations between sessions of the same mouse (intersession, *Top*) or between sessions of different mice (intersubject, *Bottom*). Quantification of intersession and intersubject similarities in the whole dataset (*Right*) revealed that intersession similarity is significantly higher than intersubject similarity. Welch’s test ***P < 0.001. (*B*) Probabilistic and (*C*) deterministic connections for the right barrel-related primary somatosensory cortex (SSp-bfd, *Top*) and for the whole brain (*Bottom*) of an individual mouse. (*D*) Tracer-based connections from SSp-bfd (*Top*) and group tracer-based SC matrix (the Allen SC, *Bottom*). (*E*) Predictive power of simulations using different types of tracer- and dMRI-based SCs. dMRI-based simulations were performed using individual or group-averaged dMRI (AVG). Welch’s test, Bonferroni corrected, **P < 0.01 ***P < 0.001. As in the following figures, the boxes, in *A* and *E*, extend from the lower (0.25, Q1) to the upper (0.75, Q3) quartile values of the data, with a line at the median. The *Upper* whisker of each box extends to the last datum less than Q3 + 1.5*(Q3 − Q1); the *Lower* whisker extends to the first datum greater than Q1 − 1.5*(Q3 − Q1).

We collected both dMRI and awake resting-state fMRI data (7 sessions per animal) from 19 hybrid B6/129P mice. We extracted SC from dMRI data to build individual virtual brains, which were imported into The Virtual Mouse Brain (TVMB), the extension of the open source neuroinformatic platform TVB (9) designed for accommodating large-scale simulations and analysis in the mouse, to generate in silico BOLD activity (21) using the reduced Wong Wang model (14, 23). We then compared simulated and empirical FC for each mouse in order to assess the power that an individual SC has to predict individual empirical FC derived from resting-state fMRI data (*SI Appendix*, Fig. S1). Further, SC was also obtained from the Allen connectome (our gold standard) in TVMB (21) to determine the contribution of information not available in dMRI-based SC. Experimental and simulated resting-state activity was characterized by a dynamical switching between stable functional configurations as revealed by the typical checkerboard patterns of functional connectivity dynamics (FCD, *SI Appendix*, Fig. S2 *A* and *B*), as observed previously (14, 24, 25). As expected, FCD varied across recording sessions (*SI Appendix*, Fig. S2*B*). In contrast, static FC was stable between experimental recording sessions (Fig. 1*A* and *SI Appendix*, Fig. S2*C*). To compare the goodness of in silico resting-state dynamics against in vivo data, we needed a metric stable across experimental recording sessions in individual subjects, and thus we used the static FC for evaluating the predictive power (PP) of a SC, instead of FCD.

We first defined the upper bound of the PP. The correlation value calculated between any pair of empirical FC for each mouse provides us with an upper boundary of the PP, taking into account intersession variability and other sources of noise that preclude 100% PP accuracy (7, 26). In keeping with human data (6, 27), we found a high intersession correlation for each of the 19 mice, demonstrating stability across different recording sessions in a given mouse (Fig. 1*A*). Intersession correlations within the same animal were greater than intersubject correlations, indicating that there is an individual functional organization per mouse, which may act as a functional fingerprint. Next, we sought to examine the extent to which individual functional connectomes correspond to individual structural connectomes.

### SC Obtained with a Deterministic Algorithm Is a Better Predictor of FC.

Here we considered probabilistic (Fig. 1*B*) and deterministic (Fig. 1*C*) dMRI-based SCs, using iFOD2 (28) and SD_Stream (29) within MRtrix3 software (29) tractography algorithms, respectively. SC obtained with the deterministic algorithm yielded a greater PP than the SC obtained with the probabilistic one ($PP_{Individual-\det}=0.415\pm 0.005$, $PP_{Individual-prob}=0.392\pm 0.005$, $mean\pm SD/\sqrt{N}$, P=0.008 for the Welch’s test Bonferroni corrected, Cohen’s *d* = 0.45, 95% CI = [0.19, 0.71], *n* = 120; Fig. 1*E*). The significative density difference in the 2 kinds of connectomes ($Density_{Individual-\mathrm{Pr}ob}=69\pm 1\%$, $Density_{Individual-\det}=28.2\pm 0.2\%$, $\text{P}=3{\text{e}}^{-20}$ for the Welch’s test, Cohen’s *d* = 12, 95% CI = [8, 17]), by itself, is not enough to explain the observed discrepancy in the PP. Connection density does not fully account for the predictive power of a connectome, but instead the relation depends on the connectome derivation (*SI Appendix*, Fig. S3). We argue that the observed difference in PP between deterministic and probabilistic processed connectomes depends on the proportion of false negative (FN) and false positive (FP) connections introduced by the 2 different algorithms. Zalesky et al. (30) show that the typical brain small-world topology is biased by the introduction of FP connections 2 times more than by the introduction of FN connections. In line with this finding, we attribute the difference in PP of the 2 connectomes to the detrimental role of FP connections, which are more likely introduced by probabilistic than deterministic tractography. However, deterministic tractography more likely overlooks some connections, introducing FN. This highlights the importance of preserving SC specificity (FN versus FP) versus SC sensitivity (FP versus FN) in the context of large-scale models. Namely, to preserve the global topology, specificity is more important as sensitivity in SC reconstruction. In the following, we compared deterministic SC-based simulated and empirical FCs.

### Individual SC Is the Best Predictor of Individual FC.

Next, we found that individual SCs had a greater predictive power than the averaged SC ($PP_{Individual-\det}=0.415\pm 0.005$, $PP_{AVG-\det}=0.377\pm 0.003$, $\text{P}=3{\text{e}}^{-9}$ for Welch’s test Bonferroni corrected, Cohen’s *d* = 0.86, 95% CI = [0.60, 1.12], $PP_{Individual-prob}=0.392\pm 0.005$, $PP_{AVG-\mathrm{Prob}}=0.349\pm 0.004$, $\text{P}=2{\text{e}}^{-11}$ for the Welch’s test Bonferroni corrected, Cohen’s *d* = 0.97, 95% CI = [0.71, 1.23], *n* = 120; Fig. 1*E*), showing the importance of individual SCs. Although the Allen SC was obtained from hundreds of different mice, we found that it had a greater PP than individual dMRI-based SCs ($PP_{Individual-\det}=0.415\pm 0.005$, $PP_{Tracer}=0.488\pm 0.005$, $\text{P}=4{\text{e}}^{-21}$ for the Welch’s test Bonferroni corrected, Cohen’s *d* = 1.39, 95% CI = [1.05, 1.72], *n* = 120; Fig. 1 *D* and *E*), suggesting that the tracer-based connectome includes structural information that is not present in dMRI, but which is central to explain the emergence of the functional connectome, even at the individual level. As the Allen SC was built from C57BL/6 mice, we verified the generality of our results in this strain (*SI Appendix*, Fig. S4*A*). Global signal regression, which improves structure–function relations and averaging recording sessions within each mouse (31), which reduces noise, increased the PP but did not alter the results (*SI Appendix*, Fig. S4 *B* and *C*). Finally, splitting the recording sessions of each mouse and submitting the data to a test–retest analysis revealed a close agreement between datasets (*SI Appendix*, Fig. S4*D*). Thus, our conclusions are strain and preprocessing independent, and reproducible.

### Importance of Long-Range Connections and Directionality.

**Fig. 2.**
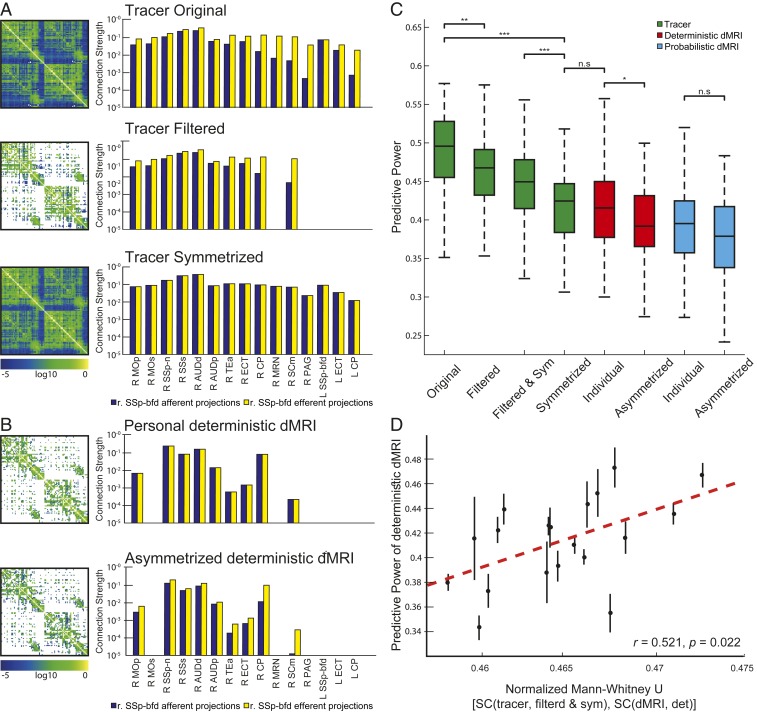
Structural connectome (SC) surrogates generated from the tracer-based SC (*Left*) and representative connections of the SSp-bfd (*Right*). The original, filtered and symmetrized SCs are shown in the *Top*, Middle, and *Bottom* rows, respectively. (*B*) Representative individual deterministic diffusion MRI (dMRI) SC (*Left*) and SSp-bfd’s connections (*Right*) before (*Top*) and after (*Bottom*) a-symmetrization. (*C*) Comparison between the performances of different surrogate SC demonstrates the effects of filtering and/or symmetrization of the Allen SC and a-symmetrization of the individual deterministic and probabilistic dMRI-based SCs. Welch’s test, Bonferroni corrected, *P < 0.05, **P < 0.01, ***P < 0.001; n.s., nonsignificant. (*D*) The relations between predictive power (PP) of filtered and symmetrized tracer SC and individual deterministic dMRI SCs are quantified through the normalized Mann–Whitney *U* statistic and demonstrate significant correlation. The greater *U* values represent greater similarity between individual deterministic dMRI SCs and the Allen SC. Brain region terms are listed in the *SI Appendix*, Table S1.

To identify the source of the systematic superior performance of the Allen SC, we focused on the major limitations of dMRI: 1) difficulty in resolving long axonal tracts, 2) lack of information on fiber directionality and synaptic transmission, and 3) imprecise estimation of connection weights caused also by the impossibility to detect synaptic connections. Although synaptic properties are not available with precision at present, other parameters can be estimated. We tested the contribution of fiber length by filtering the Allen SC to include only fibers present in the dMRI-based SC (Fig. 2*A*). We characterized the role of fiber directionality by symmetrizing the Allen SC (Fig. 2*A*), asymmetrizing the dMRI-based SC (Fig. 2*B*), and quantifying the impact of each manipulation (Fig. 2*C*).

Since dMRI fiber reconstruction reliability is inversely proportional to fiber length (16, 32, 33), dMRI SCs are sparser than the Allen SC (Fig. 1 *B*–*D* and *SI Appendix*, Fig. S3*A*). To test the influence of the missing fibers in predicting FC, we built a filtered Allen SC (Fig. 2*A*), which includes only the connections contained in at least 1 of the 19 deterministic dMRI SCs. The filtered connectome contains 32% of the connections of the original tracer connectome, which are those captured by the dMRI-based deterministic processed connectomes. The connections that remain after the filtering operation are mainly those characterized by short-range length (*SI Appendix*, Fig. S3*B*): the averaged path length of the connections in the original and filtered tracer-based connectome is 5.40 ± 0.02 mm and 3.57 ± 0.03 mm, respectively. Fig. 2*C* shows that the PP of the filtered Allen SC is lower than the original Allen SC ($PP_{Tracerfiltered}=0.461\pm 0.005$, $PP_{Tracer}=0.488\pm 0.005$, P=0.002 for the Welch’s test Bonferroni corrected, Cohen’s *d* = 0.49, 95% CI = [0.23, 0.76], *n* = 120; Fig. 2*C*); however, it remains statistically greater than the PP of individual SCs ($PP_{Individual-\det}=0.415\pm 0.005$, $\text{P}=2{\text{e}}^{-10}$ for the Welch’s test Bonferroni corrected, Cohen’s *d* = 0.92, 95% CI = [0.62, 1.20], *n* = 120; Fig. 2*C*). Thus, although connections overlooked by the dMRI method, which are mainly long-range connections, are important to explain FC, other important structural features present in the Allen SC are necessary to explain the discrepancy in PP between the tracer-based and dMRI-based connectomes.

We next focused on fiber directionality, since imposing bidirectional communication between regions connected with unidirectional links in vivo may affect FC. We used an approach based on surrogate SCs to test the role of directionality. Since the Allen SC contains directionality between regions, we removed this information by symmetrizing it (Fig. 2*A*). Fig. 2*C* shows that symmetrizing the Allen SC reduces its PP significantly ($PP_{Tracersym}=0.418\pm 0.004$, $PP_{Tracer}=0.488\pm 0.005$, $\text{P}=2{\text{e}}^{-20}$ for the Welch’s test Bonferroni corrected, Cohen’s *d* = 1.36, 95% CI = [1.02, 1.68], *n* = 120; Fig. 2*C*), making it comparable to the PP of the dMRI-based SCs (P=1.0 for the Welch’s test Bonferroni corrected, Cohen’s *d* = 0.06, 95% CI = [−0.19, 0.32], *n* = 120). This demonstrates that directionality is a key determinant of FC. It is notable that symmetrizing the filtered Allen SC led to a more modest reduction of the PP than the symmetrization of the original Allen SC ($PP_{Tracersym}=0.418\pm 0.004$, $PP_{Tracerfilteredsym}=0.446\pm 0.004$, $\text{P}=4{\text{e}}^{-5}$ for the Welch’s test Bonferroni corrected, Cohen’s *d* = 0.62, 95% CI = [0.36, 0.87], *n* = 120; Fig. 2*C*). We argue that the PP difference can be explained by considering the amount of false positive introduced in the surrogate connectomes by the transformation: the filtering operation inserts FN connections, while the symmetrization operation inserts both FN and FP connections (34). It follows that the symmetrized and filtered connectome contains less FP than just the symmetrized connectome. Thus, as previously discussed for the tractography processing, introducing FP connections, as produced by the symmetrization but not by the filtering, is more detrimental than the introduction of FN connections. To summarize when the tracer-based connectome is manipulated in order to remove the information not detected by dMRI, which is the inability to detect 1) the directionality of brain connections and 2) some brain connections, especially the long-range ones, we found that the removal of the directionality information biases the predictive power of the connectome more than the removal of the connections not detected by the dMRI method.

We then took the complementary approach: enriching the dMRI-based SC with information on fiber directionality, i.e., asymmetrizing it. The results show that asymmetrizing the dMRI SCs does not increase, but rather decreases the PP ($PP_{Individual-\det}=0.415\pm 0.005$, $PP_{Individual-\det-asym}=0.394\pm 0.005$, P=0.02 for the Welch’s test Bonferroni corrected, Cohen’s *d* = 0.42, 95% CI = [0.16, 0.67], *n* = 120; $PP_{Individual-prob}=0.392\pm 0.005$, $PP_{Individual-prob-asym}=0.377\pm 0.005$, P=0.3 for the Welch’s test Bonferroni corrected, Cohen’s *d* = 0.29, 95% CI = [0.04, 0.55], *n* = 120; Fig. 2 *B* and *C*). We argue that the asymmetrization of the dMRI connectomes biased the PP because asymmetrizing a matrix is an ill-posed problem, since there is no unique solution (more details can be found in *Materials and Methods*). In addition, there is no 1:1 correspondence between the connection strengths obtained with dMRI (axonal bundles) and Allen ones (axonal branches) since axons tend to branch more or less profusely when reaching their target zone, a feature that cannot be detected by dMRI.

### Connection Strengths as Key Determinants of FC.

The symmetric filtered Allen SC and the deterministic dMRI SCs have a similar structure: both matrices are symmetric and contain the same number of elements. Since the PP of the symmetric filtered Allen SC is still greater than the dMRI one, the difference can only result from dissimilarities in the values of the matrices’ entries, i.e., the connection strength values. Fig. 2*D* shows that there is a significant relation between the normalized U statistics of the Mann–Whitney *U* test calculated between the filtered symmetric Allen SC and the individual dMRI SC and the PP of the latter (*r* = 0.52, *P* = 0.02). Namely, the more the distribution of connection strengths of the deterministic dMRI is similar to that of the Allen SC, the more reliable the predictions are.

### Specific Refinement of Individual dMRI Connectomes.

**Fig. 3.**
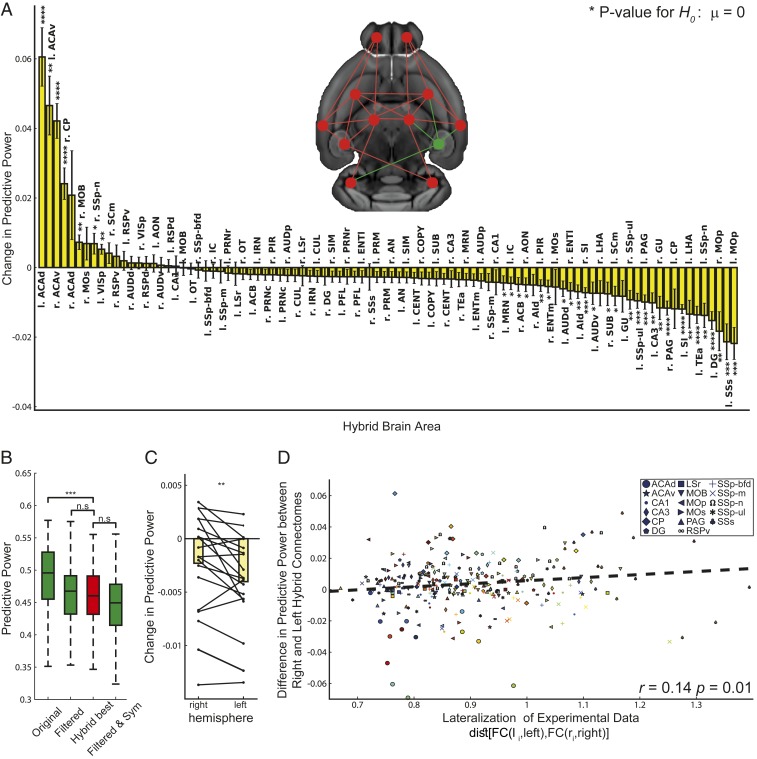
(*A*) An illustration of a hybrid SC. The connections between all areas are extracted from dMRI data (red nodes and links) except for the connections of a single area, which are obtained from the Allen SC (green). The graph shows the difference in PP between hybrid and individual deterministic dMRI SCs. The change in PP is calculated as the difference between the PP of the hybrid SC and the individual dMRI SC averaged across all sessions and animals. *P* values refer to the *t* test for the null hypothesis that substituting the tracer connections of a given brain region in the dMRI connectome does not change the PP of the connectome. Nomenclature and abbreviations are listed in *SI Appendix*, Table S1. (*B*) Comparison between the PP of tracer-based and hybrid SCs revealed that connectomes (hybrid best), which were generated by replacing the connection of a single area in each mouse, predict experimental FC better than the filtered and symmetrized connectomes (filtered & Sym). Welch’s test, Bonferroni corrected, **P < 0.01, ***P < 0.001, ****P < 0.0001; n.s., nonsignificant. (*C*) Comparison between the change in PP following hybridization of left and right brain areas reveals that replacement of left area connections decreased the PP more than right area connections (paired *t* test). (*D*) The differences in PP between right and left hybrid SCs were correlated with the lateralization of experimental FC, which was quantified as the Euclidean distance between left and right functional connections. The closer the lateralization index is to 1, the more similar are the intrahemispheric left and right area connections. Different colors label different mice.

Since some afferent and efferent connections of specific areas may not be reliably reconstructed with dMRI, we examined whether refining dMRI SCs with more precise patterns derived from the Allen SC would improve the PP. For each deterministic dMRI SC, we substituted the nonzero incoming and outgoing connections of a specific region with the corresponding Allen SC projections, thus building a hybrid connectome (Fig. 3*A* and *SI Appendix*, Fig. S5).

When considering all mice, we found that substituting the dorsal and ventral anterior cingulate areas (ACAd, ACAv) and the right caudoputamen (CP) connectivity patterns with the Allen SC projections significantly improved the PP of the connectome (left ACAd, improvement = 0.047 ± 0.006, *t* = 7.23, $\text{P}=7{\text{e}}^{-6}$ for the *t* test; left ACAv, improvement 0.032 ± 0.006, *t* = 4.96, P=0.002 for the *t* test; right ACAv, improvement = 0.028 ± 0.003, *t* = 7.58, $\text{P}=1{\text{e}}^{-4}$ for the *t* test; right CP, improvement = 0.018 ± 0.003, *t* = 6.42, $\text{P}=5{\text{e}}^{-6}$ for the *t* test; Fig. 3*B*), suggesting that both regions are poorly resolved by dMRI in mice. Importantly, the majority of substitutions decreased the PP (Fig. 3*B*).

For each individual SC, we identified the region in which replacement of its dMRI connections with the Allen ones generates a new connectome, hybrid^best^, which has the best PP improvement as compared to the other hybrid connectomes (*SI Appendix*, Fig. S5). Fig. 3*C* shows that the PP achieved by hybrid^best^ is statistically indistinguishable from the one achieved by the filtered Allen SC (P=1.0 for the Welch’s test Bonferroni corrected, Cohen’s *d* = 0.008, 95% CI = [−0.25, 0.25], *n* = 120). In other words, it is sufficient to replace in the dMRI SC the connections of 1 particular region with the corresponding Allen ones, to get a similar prediction, which is specific for each mouse.

### The Asymmetric Mouse Brain.

Finally, we sought to estimate the potential contribution of asymmetric transhemispheric connectivity. Fig. 3*D* shows that there is a considerable improvement in the PP of hybrid SCs when using connections from the right hemisphere, as compared to those from the left one. The Allen connections have been estimated using unilateral injection in the right hemisphere (18). Since no tracer injections were done in the left hemisphere, TVMB uses a mirror image of the right hemisphere to build the left one (21). This suggests that the tracer-based intrahemispheric connectivity predicts better right intrahemispheric functional behavior than the left one, as demonstrated in *SI Appendix*, Fig. S6*A*. Fig. 3*E* shows that there is a significant relation between hemispheric lateralization in the functional connectomes and the improvement in PP when the right and left homotopic tracer area’s connections are introduced in the dMRI SC (r=0.14,P=0.01). Namely, the more functional connections are asymmetric, the more the PP decreases when using the right hemisphere connections to build the left ones. These results suggest that connectivity asymmetry impacts brain dynamics and that it is region and mouse specific.

### Hemispherical Lateralization of the Mouse Brain.

**Fig. 4.**
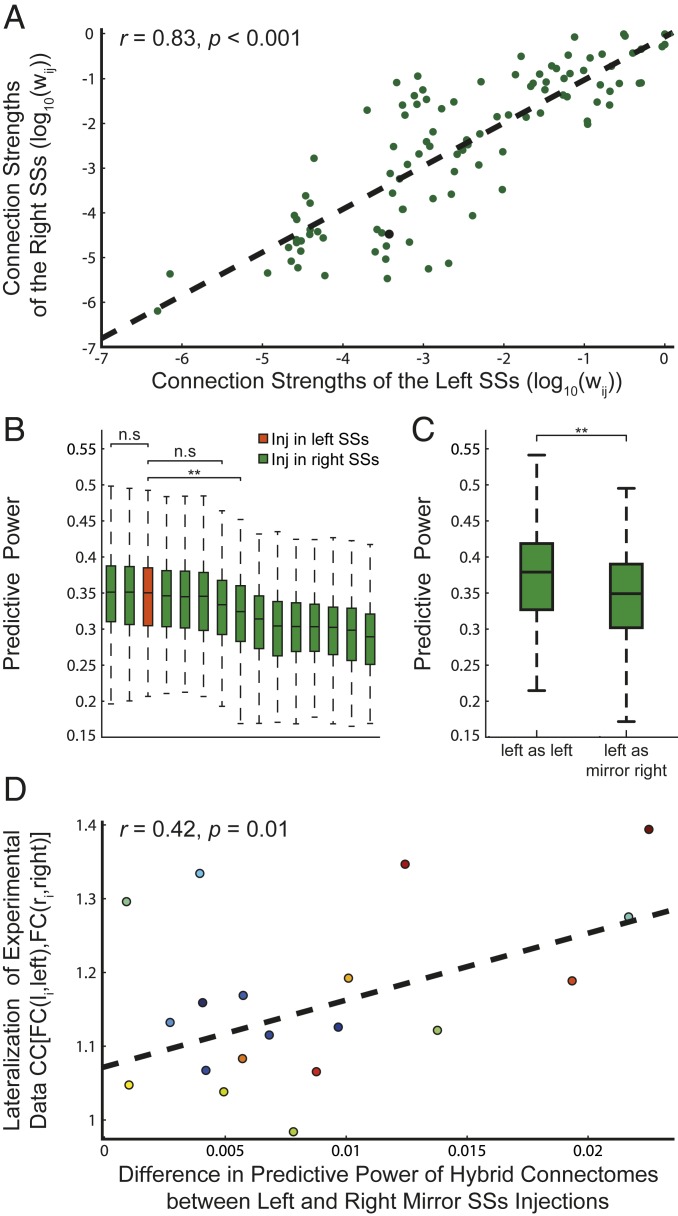
(*A*) Relation between fiber projections of the left and right SSs. (*B*) The height of the bars represents the predictive power of tracer-based connectomes built using just 1 injection experiment per area. This procedure differs from the general tracer building procedure used in this work since generally the connections of each region are calculated as the average of the results of all injection experiments performed in that region. The height of the orange bar is the predictive power of the tracer-based connectome whose left SSs connection are reconstructed from the tracer injection experiment in the left SSs. The green bars are the predictive power of the tracer-based connectomes whose left SSs connections are built as the mirror image of the connections measured from each of the 14 experiments performed in the right SSs area. The statistical difference between the bars is assessed through the Welch’s test, Bonferroni corrected, **P < 0.01; n.s., nonsignificant. (*C*) Comparison between the predictive power of hybrid connectomes built as a dMRI-based connectome with left SSs tracer connections reconstructed from the tracer injection experiment in the left SSs or as the mirror image of right tracer connections retrieved from the right SSs experiment whose injection coordinates are closer to those of the left SSs injection experiment. (*D*) The differences in PP between true left and mirror right SSs hybrid SCs are correlated with the lateralization of experimental FC.

Fig. 3*E* shows that the region demonstrating the greatest lateralization in terms of functional connectivity in individual mice is the supplemental somatosensory area (SSs). Fig. 3*B* shows that when we introduce the mirror image of the right SSs into the dMRI SC, the predictive power is considerably decreased, which means that the mirror image of the right SSs poorly represents the true left SSs. We thus focused on the SSs area. If SC drives FC, we predicted that introducing in the tracer-based connectome the detailed left SSs connections, instead of using the mirror image of the right SSs ones, would increase the PP of the connectome. We first performed tracer injections in the left SSs and determined the projection pattern. As predicted, we found evidence of an asymmetric distribution of fibers between the left and right SSs (Fig. 4*A*). To test whether these structural differences were sufficient to explain the functional ones, we introduced the connections of the left SSs into the tracer connectome and obtained a statistically greater PP as compared to the ones of purely mirrored connectomes built from the injection experiments performed in the right SSs (Fig. 4*B*). Next, we introduced the left connections of the SSs into the dMRI-based SCs (hybrid connectome), and, as predicted, we found a greater PP as compared to using the mirror image of the right connections of the SSs as shown in Fig. 4*C* (between the 14 experiments performed in the right SSs we consider the one whose injection location is more similar to those used in the left SSs injection experiment). Finally, since our previous results demonstrate that the lateralization is animal dependent, we sought to examine whether lateralized FC is supported by lateralized SC and found that the improvement of the PP following hybridization of left SSs dMRI connections is indeed proportional to the degree of functional lateralization (Fig. 4*D*). Together, these results show that the mouse brain is structurally lateralized, and that this lateralization impacts whole brain dynamics at the individual subject level.

## Discussion

Our results provide direct evidence of causality between SC and FC, in the sense that individual structural connectomes predict their functional counterparts better than the dMRI-based averaged connectomes. The causal link is established by joining individual structural connectomes with mechanistic models to create dynamic virtual brain models, capable of generating neural source signals and their corresponding functional brain imaging signals. The emergent spatiotemporal dynamics of the virtual brain model is constrained by the structural connectome, but it has been unclear so far if interindividual structural variability causes sufficient influence on the functional level and if these functional variations can be reliably captured by the metric of FC. As both biological systems and their mechanistic models show sensitivity to noise and display nonstationary dynamics and multiscale behavior, the causal relation of SC and FC may have been lost in the complexity of the measured brain imaging signals. We have here demonstrated that this is not the case. Previous studies utilized the Allen Mouse Connectivity Atlas to study structure–function relations at the group level using voltage-sensitive dyes (35) and FC (6, 22, 36). In addition, a recent work in rats (37) used TVB to simulate FC based on SC and found a strong correlation at the group level. A similar finding has been reported in humans (38). Here we compared structure–function relations in individual mouse brains and we used the detailed Allen connectome as a gold standard to identify regions and connections that play a preeminent role in the emergence of individual brain dynamics. We showed that, similar to humans (6), intramice FCs are more stable than intermice FCs (Fig. 1*A*). We propose that the emergence of individual features in the functional data are, at least partially, driven by the individual’s structural connectivity with stable features encoded in the connectome (Fig. 1*E*). In addition to connectome specificity, other structural data features may drive functional intersubject variability, including foremost regional variance such as synaptic receptor type and density (39), but also methodological variations such as parcellation differences (3). Notwithstanding, we cannot exclude that the variations in hemodynamic response functions (HRFs) across animals and brain location affect SC–FC relations, as it has been shown to contribute to individual variability in human FC estimation (40). In this study we aimed at reducing this variability by scanning awake mice, reducing the confounding effects of anesthesia, and allowing collection of data over multiple sessions per mouse (41, 42). Moreover, we used spin-echo echo planar imaging (EPI), which is more sensitive to microvasculature relative to gradient-echo EPI, especially at high magnetic fields (43), further reducing the variability of HRF (40). Finally, HRF variability can increase the number of false positives in FC in empirical data, but it cannot explain differences in predictive power of simulated data obtained from different structural connectomes.

Virtualizing different structural connectomes, we found that group tracer-based connectomes predict empirical FC better than individual dMRI-based connectomes, and while we argue that this difference can be explained by better measurements of long-range connections, fiber directionality and connection weights, we cannot rule out that it is caused, at least partly, by the compromised diffusion tensor imaging (DTI) data quality of in vivo measurements. As compared to ex vivo studies (44), we designed our DTI sequences with relatively low numbers of gradient directions, anisotropic voxels, and relatively low resolution. Together with motion-related noise, these factors reduce the quality of tractography (45) and may contribute to the lower performance of dMRI-based SC in predicting empirical FC. Nevertheless, the DTI sequence was designed this way to allow in vivo measurement in mice which is comparable to human data which will support future mechanistic investigations of SC–FC relations. The dMRI–connectome reconstruction could be improved by using more sophisticated anatomical constraints in the tractography pipeline (e.g., anatomically constrained tractography [ACT] method, ref. 46) in addition to the basic segmentation in regions of interest.

The detrimental role of FP connections in the connectome topology has been previously explored by refs. 30 and 34, analyzing, respectively, the effect of FP as introduced by probabilistic tractography and overlooking the connections directionality. In line with these findings, we showed that the introduction of FP connections biases the connectome predictions. We found the dMRI-based connectomes processed with the deterministic tractography have a statistically greater PP than those processed with probabilistic algorithms. Since the observed difference in PP is not directly related to the difference in connections density (*SI Appendix*, Fig. S3), we argue that the difference in PP is driven by the different characteristics of the connections overlooked by both types of tractography processing: more FP and less FN in the case of probabilistic processed connectivity, and conversely, in the case of deterministic processed connectivity. This highlights that brain dynamics predictions are more accurate if connectome specificity is preserved, even at the cost of sensitivity, as it is the case of deterministically processed connectomes.

When processing the tracer-based data, the probabilistic computational model used to construct the original Allen connectome (18) may introduce several false negative connections, resulting in a low connection density reconstruction (35 to 73%), while others reported a 97% density (19, 20). Here, we have used the Allen connectome builder interface, which implements a deterministic approach to reconstruct whole brain connectivity (21), leading to a 98% density of connections. Still, as shown in Fig. 2*B*, the introduction of FN connections (filtered tracer-based connectome) does not dramatically influence the PP of the connectome.

The main drawback of the Allen connectome is that it has been obtained from hundreds of different mice, thus blurring individual variability. We found that replacing most individual dMRI connections with Allen connections reduces the PP. However, in some regions such as the anterior cingulate and the caudoputamen, group-level Allen connections outperform individual dMRI connections. This finding can be explained by the fact that connections from the anterior cingulate are difficult to resolve, as this area is located in the midline brain region, where the cortex folds, resulting in an abrupt change in fiber directionality. Moreover, the axons make sharp turns around the corpus callosum while the extraction algorithm assumes a systematic continuation of the vector direction. The connections of the striatum are often short and, due to its multipolar organization, without a clear gradient orientation limiting fiber reconstruction. These considerations apply to mouse strain used here. They cannot be translated as such to other species. But the conceptual framework we introduce shows how to take into account detailed connectivity patterns (if available) to improve the analysis of the SC–FC relationship. In humans, the release of full connectomes obtained postmortem at ultrahigh resolutions (47) constitutes an important step in this direction. Detailed connectivity patterns from nonhuman primates may also be used to build a high resolution “human” SC (48, 49).

Although the Allen connectome was obtained from C57BL/6 mice, brain dynamics of hybrid F1 mice could be predicted by the Allen connectome, suggesting that the structural organization of the mouse brain was not impacted by outbreeding. Findings from hybrid mice are considered more generalizable to other strains (50), thus suggesting that the pattern observed here is not strain specific. Nonetheless, since the genetic background affects the behavioral phenotype (51), it will be important to systematically assess these findings in mouse strains where this aspect is directly manipulated.

The Allen SC includes directionality and long-range connections, which are not well (or at all) resolved by dMRI. However, the removal of the connections not resolved by dMRI-based connectomes, mostly those characterized by long-range length, is not sufficient to explain the discrepancy between the tracer-based and dMRI-based predictive power. In addition, we showed that removing the directionality information from the tracer-based connectome, that it is symmetrizing the connectome, thus introducing FP and FN connections, worsens the predictive power more than the filtering operation, that consist in introducing just FN connections (34). This shows the key role of connections directionality in predicting brain dynamics; and it confirms our results on tractography algorithm processing: FP connections biases the predictive power ability of the connectome more than FN. Finally, analyzing the connection strength differences between the dMRI and tracer-based connectome, we have shown that connection strengths are the main determinant of these dynamics, and consequently of individuality (Fig. 2*D*).

An unexpected result was the important role played by the transhemispheric asymmetry of connections. This finding is consistent with calcium imaging studies reporting such asymmetry in rodents (52). By comparing injections between left and right hemispheres, we confirmed our prediction that the approximation of left area connections as compared to right area connections, necessary in the tracer-based connectome reconstruction, significantly affect the predictive power of the connectome. Moreover, we showed that the bias introduced by this approximation is proportional to the degree of the individual animal’s functional lateralization.

We quantified PP using the linkwise Pearson correlation across experimental and simulated FC. This choice of metrics has limitations linked to the stability of the functional data features during the time window considered. Other choices would have been possible, as for example comparing specific features of the FC (e.g., functional hubs, graph theoretical characteristics, subnetwork structures, etc.) or evaluating the dynamical evolution of the functional links, e.g., FCD or functional meta connectivity (FMC) (*Materials and Methods*). Our definition of the PP, broadly accepted in literature (12–14, 37, 53–56), responds to the need to quantify the ability of a model to reproduce the functional brain behavior globally, and not its specific features. However, overlooking the informative content encoded in the dynamics of the FC is a limitation of our study. FMC is 1 means of quantifying the FCD globally via its dynamics of the functional links, but has proven to be too variable across resting state sessions within the same animal (*SI Appendix*, Fig. S2). This variability limits the possibility to compare simulated and experimental FMC and to use it as a PP metric. Although we are omitting FCD features from the PP evaluation, these features are integral to the simulations (*SI Appendix*, Fig. S2).

Another limitation of our work is the assumption of region invariance, that is we use the same model, as well as the same parameters, to describe the activity of all of the brain regions, both cortical and subcortical ones. The only symmetry breaking between the virtual brain areas is their integration in the network, i.e., their anatomical connections. It follows that in this approximation the connectome acquires a central role. This centrality allows us to trace back all of the prediction differences obtained in the virtual mice to the connectome used, as it is our aim. However, we do acknowledge the need to introduce regional specificity and variance into the brain models. Future evolutions of large scale brain models should include such specificities.

Progress in connectomics enabled the development of large-scale brain models to study brain function in health and disease (12, 57). Although individual whole brain modeling has a potentially high translational value for the benefit of patients (15, 58, 59), the entire approach relies on the extent to which individual differences in structural connectomes determine the emergent network dynamics and consequent neuroimaging signals. Although SC does not provide enough information to predict an epileptogenic zone in humans (60), our work shows that using more precise information (e.g., obtained from tracer injections in nonhuman primates) to consider directionality, synaptic weights and poorly resolved dMRI connections, will increase the predictive power. As for clinical applications, the value of a generalized model is measured by its utility in individuals (15, 57); our results bear a significant promise in this domain as they demonstrate and quantify the predictive capacity of SC and FC variability.

In conclusion, we identified key individual structural features (fiber directionality, connection strength and patterns, and interhemispherical asymmetry), which are relevant to predict the emergence of the functional patterns during a resting state in mice. Our results strongly suggest the existence of a causal relation between the structural and the functional connectome. Although the detailed structural results presented here are species specific, our conceptual framework is species invariant and can now be exploited in humans for individual diagnosis and clinical decision making.

## Materials and Methods

Additional details on materials and methods are provided in *SI Appendix*. All procedures were conducted in accordance with the ethical guidelines of the National Institutes of Health and were approved by the Institutional Animal Care and Use Committees (IACUC) at Technion and Allen Institute for Brain Science. Mice were group housed to prevent stress (61).

### Structural and Functional Experimental Data.

Nineteen male hybrid nonanesthesized mice were scanned using a 9.4 Tesla MRI to obtain structural information and resting state functional data, 6.31 ± 0.82 (mean ± SD) sessions of 15.7 ± 4.4 min length (mean ± SD) per mice. The structural data have been processed using using MRtrx3 software (29). To obtain the tract streamlines, we integrated the field of orientation probability density using both deterministic (SD_Stream, ref. 29) and probabilistic (iFOD2, ref. 28) algorithms in order to obtain, respectively, deterministic and probabilistic processed dMRI-based connectome.

The tracer-based connectome was built through the Allen Connectivity Builder pipeline (21), implemented in The Virtual Brain software (9). The pipeline allowed the manipulation of the anterograde tracer experiments performed at the Allen Institute (18) to reconstruct a tracer-based mouse connectome and related brain volume.

### Surrogate Connectomes.

In order to test different hypotheses about what could be the connectivity properties that give rise to the observed discrepancies in the simulated dynamics, we built different kinds of surrogate connectomes: a dMRI-based averaged connectome (averaging the 19 dMRI based connectomes), a filtered tracer-based connectome (filtering out the connections not detected in the dMRI connectomes), a symmetrized tracer-based connectome, asymmetrized dMRI-based connectomes, and hybrid connectomes (dMRI-based connectomes whose connections of 1 area are replaced with the tracer-based connections of that area).

### Simulate Resting State Dynamics.

We simulate resting state dynamics using the connectome-based model approach as implemented in The Virtual Brain software (9, 62). In particular, we used the reduced Wong Wang model (13, 23) in the bistable configuration in order to reproduce the dynamical switching of the functional connections (14). We transformed the simulated synaptic activity in BOLD signal using the Balloon-Windekessel method (56, 57).

### Analysis.

The emergence of the functional organization in the experimental and simulated brains was analyzed through the static FC, the FCD, and the FMC.

We quantified the PP of a given connectome *c* as the Pearson correlation between the simulated FC, obtained using that connectome *c,* and the FC arranged during resting state experimental recordings.

In order to assess the significance of the difference in PP of differently derived connectomes we used the *P* value calculated through the Welch’s test; we corrected the *P* values for multiple comparisons using the Bonferroni correction. We measure the effect size using the Cohen’s *d* and we calculated the 95% CIs using the estimation stats framework as described in ref. 63 and available at https://www.estimationstats.com/.

### Data Sharing.

We used The Virtual Brain (https://www.thevirtualbrain.org/tvb/zwei), an open access platform, for the simulations. It includes all pipelines for importing empirical data (e.g., structural data from the Allen Institute) and all analyses/display routines. All imaging raw data and the relevant codes used in this study are available in BIDS format on OpenNeuro, https://openneuro.org/datasets/ds002307.

## Supplementary Material

Supplementary File
