# Supplementary material for: Individual structural features constrain the mouse functional connectome
Source: Proc Natl Acad Sci U S A. 2019 Dec 11;116(52):26961–9. doi: 10.1073/pnas.1906694116 (PMC6936369; doi:10.1073/pnas.1906694116)
Supplement: Supplementary File [file pnas.1906694116.sapp.pdf]

SI APPENDIX

1. Animals and Surgical Procedures

All procedures were conducted in accordance with the ethical guidelines of the National Institutes of Health and were approved by the institutional animal care and use committee (IACUC) at Technion. 19 male first generation hybrid mice (B6129PF/J1, 9-12 weeks old) were implanted with MRI compatible head-posts using dental cement as previously described (1). After 3 days of recovery, the animals were acclimatized to extended head fixation. This training included 5 handling sessions performed over 3-5 days, and 4 daily acclimatization sessions inside the MRI scanner. In each acclimatization session, mice were briefly anesthetized with isoflurane (5%), and then head-fixed to a custom-made cradle for gradually longer periods (2, 5, 10, 25 min). Subsequently, mice underwent seven 45 min long awake imaging sessions, and one diffusion tensor imaging (DTI) session under continuous isoflurane anesthesia (0.5-1%). A second group that included 7 male inbred C57BL/6 mice (11-16 weeks old) was operated and scanned according to the same protocol. Mice were group housed to prevent social isolation-induced stress (2).
Experiments involving mice were approved by the Institutional Animal Care and Use Committees of the Allen Institute for Brain Science in accordance with NIH guidelines. For left side injections into SSs, surgical procedures were followed as described in (3). In brief, a pan-neuronal AAV expressing EGFP (rAAV2/1.hSynapsin.EGFP.WPRE.bGH, Penn Vector Core, AV-1-PV1696, Addgene ID 105539) was used for injections into wildtype C57BL/6J mice at postnatal day 56 (stock no. 00064, The Jackson Laboratory). SSs was targeted using stereotaxic coordinates from Bregma (AP: -0.7, ML, -3.4 and -3.9) and from brain surface (DV: 1.66). rAAV was delivered by iontophoresis with current settings of 3 μA at 7 s 'on' and 7 s 'off' cycles for 5 min total, using glass pipettes (inner tip diameters of 10–20 μm). Mice were perfused transcardially and brains collected 3 weeks post-injection for imaging using serial two-photon tomography, using methods as previously described for the Allen Mouse Connectivity Atlas (3).

2. *Data acquisition (fMRI and diffusion-MRI)*

MRI scans were performed at 9.4 Tesla MRI (Bruker BioSpin GmbH, Ettlingen, Germany) using a quadrature 86 mm transmit-only coil and a 20 mm loop receive-only coil (Bruker). Mice were shortly anesthetized (5% isoflurane) before mounted on the cradle. After acquisition of a short low-resolution rapid acquisition process with a relaxation enhancement (RARE) T1-weighted structural volume (TR = 1500 ms, TE = 8.5 ms, RARE-factor = 4, FA = 180°, 30 coronal slices, 150 × 150 × 450

www.pnas.org/cgi/doi/10.1073/pnas.1906694116

μm^3 voxels, no interslice gap, FOV 19.2 × 19.2 mm^2, matrix size of 128 × 128), four spin echo EPI (SE-EPI) runs measuring BOLD fluctuations were acquired (TR = 2500 ms, TE = 18.398 ms, 200 time points, FA = 90°, 30 coronal slices, 150 × 150 × 450 μm^3 voxels, no interslice gap, FOV 14.4 × 9.6 mm^2, matrix size of 128 × 128). In addition, mice underwent another session under anesthesia to acquire high resolution T2 image (TR = 6000 ms, TE = 8.8 ms, RARE-factor = 16, FA = 180°, 36 coronal slices, 100 × 100 × 400 μm^3 voxels, FOV 16 × 16 mm^2, matrix size of 160 × 160, 10 averages) and diffusion tensor imaging data (DTI) with a diffusion-weighted spin-echo echo-planar imaging (EPI) pulse sequence (TR = 9000 ms, TE = 21.68 ms, $\Delta/\delta = 11/2.6$ ms, 4 EPI segments, 30 gradient directions with a single b-value at 1000 s/mm^2 and three images with b-value of 0 s/mm^2 (B0), 36 slices, 100 × 100 × 400 μm^3 voxels, FOV 16 × 16 mm^2, matrix size of 160 × 160, 2 averages). Each DTI acquisition took 39.6 min.

3. Data processing

Intrinsic functional connectivity data:

fMRI data preprocessing procedure was validated in a previous study (1). Briefly, the first two time points were removed for T1-equilibration effects, slice-dependent time shifts were compensated, head motion was corrected using rigid body correction, volumes were registered to a downsampled version of the Allen Mouse Brain Atlas, and data underwent intensity normalization. Then, motion scrubbing procedure was applied to remove motion-related artifacts as previously shown. Rigorous censoring criteria were used including frame displacement (FD) of 50 μm and temporal derivative root mean square variance over voxels (DVARS) of 105% of median. An augmented temporal mask of 1 frame before and 2 frames after detected motion was used and sequences of less than 5 included frames were also censored. Runs with less than 50 frames, and sessions with less than 125 frames (5.2 mins) were excluded. The average number of included sessions per mouse was 6.31±0.82 (mean±SD) for the F1 hybrid mice and 3.71±2.21 for the C57BL/6 inbred mice. Total included time per session was 15.7±4.4 (minutes per session, mean±SD) and 11.41±3.67, respectively.

After motion scrubbing, resting-state fMRI specific preprocessing procedure was applied including demeaning and detrending, nuisance regression of 6 motion axes, ventricular and white matter signals and their derivatives, temporal filter (0.009 < f < 0.08 Hz), and spatial smoothing (Gaussian kernel with FWHM of 450 μm.) The C57BL/6 group was preprocessed both with and without global signal regression to test the effects of this procedure on structure-function relations.

To estimate functional connectomes, we build a parceled volume with a resolution compatible with the fMRI technical constraints by manipulating the Allen Mouse Brain Connectivity Atlas (3) downloaded through The Virtual Brain (4, 5). The volume was registered to the space of the functional data ('target.nii.gz') using the nearest neighbor interpolation (FLIRT software, (6)). The parcellation was reduced only to the areas where the SNR was higher than 12, and that had a volume greater than 10 voxels (>0.1mm^3). Finally, very anterior and posterior areas, such as the main olfactory bulb and cerebellum, were excluded from the parcellation due to registration problems and susceptibility artifacts associated with the head-post implantation. Once the parcellation volume was built, mean BOLD signals were extracted from the voxels composing each parcel, and correlations were calculated from included frames only (based on motion scrubbing).

Diffusion-MRI data:

We processed diffusion-MRI data using MRtrix3 software (7).

The fiber orientation distribution of each voxel was estimated using the Constrained Spherical Deconvolution (CSD, (8)). To obtain the tract streamlines we integrated the field of orientation probability density using both deterministic (SD_Stream, (7)) and probabilistic (iFOD2, (9)) algorithms; in both cases, the tracts number was set to 100 million. The streamlines were then filtered using the SIFT algorithm (10) which selectively reduces the number of tracts exploiting the fiber orientation density information obtained through the CSD in the previous step. The filtered tracts of the right SSp-bfd obtained with probabilistic and deterministic algorithm, for an illustrative mouse, are shown in Figure 1B and 1C respectively. We defined seed regions using the Allen Mouse Brain Connectivity Atlas (3) obtained through the The Virtual Brain (4, 5); after registering the volume in the individual mouse diffusion space, we reduced the parcellation only to those areas whose volume was greater than 250 voxels (>1.125mm^3).

Using the deterministic and probabilistic streamlines and the node parcellation image, we generated a connectome. The connection strength between each pair of nodes was defined as the streamline count between the two nodes scaled by the inverse of the volumes of the two areas. A radial research was performed to assign each streamline end point to a given node. If no node was found inside a sphere of 1 mm radius, the streamline was not assigned to any node. We excluded all self-connections by setting the diagonal elements of the connectome to zero and normalized all connection strengths between 0 and 1. Then, we repeated this procedure for all mice. An example of personalized connectome obtained with probabilistic and deterministic algorithm is shown in Figure 1B and 1C, respectively.

Tracer data:

The recent updates of The Virtual Brain software (4, 5) allows us to manipulate the anterograde tracer experiments performed at the Allen Institute (3) in order to obtain a very precise mouse connectome. Specifically, we define the link between two brain regions according to the anterograde tracing information provided by the Allen Institute of Brain Science and presented in the work of Oh et al., 2014 (3). In the latter, the axonal projections from a given region are mapped by injecting in adult male C57Bl/6J mice the recombinant adeno-associated virus, which expresses the EGFP anterograde tracer. The tracer migration signal is detected with a serial two-photon tomography system. This approach is repeated systematically in order to collect the information on the tracer migration from several injection sites in the right hemisphere to target regions in both ipsilateral and contralateral hemispheres; for each injection sites several experiments are run and distinct measures are accomplished. Through the Allen Connectivity Builder interface in TVB we define the connection strength between source region i and target region j as the ratio between the projection density (the number of detected pixels in the target region normalized on the total number of pixels belonging to that region) and the injection density of the source region (the number of infected pixels in the source region normalized on the total number of pixels belonging to that region). The TVB pipeline, once downloaded the raw data, i.e. projection density and injection density for experiment, from the Allen dataset, manipulate the data in order to build a connectome as described in Melozzi et al., 2017 (5).

Unless otherwise specified, the tracer-based connectome is built averaging experiments performed injecting the tracing compound in the areas in the right hemisphere.

One of the main differences between tracer and diffusion-MRI technique is the spatial resolution; in order to discard this factor as a cause of diversity in the reconstructed connectome, the seed areas included in the tracer connectome are the same as the ones included in the diffusion-MRI connectome. As for the diffusion-MRI connectome, the self-connections were excluded and the connection strengths were normalized between 0 and 1. The tracer connectome is shown in Figure 1D.

To evaluate the impact of introducing connections of the left SSs obtained injecting the tracing compound in the left structure (and not in the right structure as generally done in the building procedure) we built tracer-based connectome using the information of just one experiment per area (Figure S6b). In particular in Figure 4B we evaluate how reconstructing left SSs connections using different experiments (14 injection experiments performed in the right SSs and 1 injection

experiment in the left SSs) impact the Predictive Power of the tracer connectome.

4. Surrogate connectomes

Connectomes derived with different methodologies (e.g. tracer experiments, deterministic or probabilistic diffusion-MRI tractography) give rise to different simulated resting state dynamics. Since in this study we always use the same large-scale model to simulate the functional brain patterns (reduced Wong Wang model in the bistable configuration, see section simulated dynamics), the observed differences are determined uniquely by the different structural organization used to conceptualize the brain network, i.e. the connectome.

In order to test different hypotheses about what could be the connectivity properties that give rise to the observed discrepancies in the simulated dynamics, we built different kinds of surrogate connectomes as described in what follows.

Averaged connectome: the role of individual variability

In order to assess the role of individual variability in dMRI data, we built an averaged connectome, both for deterministic and probabilistic tractography. We defined the averaged connectome as a matrix whose entry $w_{ij}^{'}$, i.e. the connection strength between area *i* and area *j*, is the arithmetic mean of the values of the connection strength w_{ij} of the N individual dMRI connectomes containing both area *i* and area *j*:

$$w_{ij}^{'} = \frac{1}{N}\sum_{n=1}^{N} w_{ij}{}^{n} \quad (1)$$

where *n* is the connectome index.

Filtered connectome: the role of long-range connections

Comparing the connectomes in Figure 1B-D it is possible to notice that the number of long-range connections detected with probabilistic, and more dramatically with deterministic, tractography is drastically lower than the one retrieved with the tracer method. It is well known that the accuracy of fiber reconstruction with diffusion-MRI data decreases with fiber distance; however, it is still unclear how to address this methodological limitation.

In order to quantify the impact of long-range connections presence in the simulated system, we filtered down the tracer connectome by removing all the connection not present in the deterministic diffusion-MRI connectomes. The filtered tracer connectome is shown in Figure 2A.

Symmetrized and asymmetrized connectome: the role of fiber directionality

The incapacity to detect fiber directionality is one of the main drawbacks of dMRI method.

In order to understand the influence of this property in the simulated system, we symmetrized the tracer connectome and we asymmetrized the diffusion-MRI connectome.

Symmetrized tracer connectome:

For each asymmetric matrix exists one, and only one, decomposition that enables us to find the corresponding symmetric matrix: each generic matrix A can be decomposed in its symmetric and asymmetric part as:

$$A = A^{\text{sym}} + A^{\text{asym}} = \underbrace{\tfrac{1}{2}(A + A^T)}_{\text{symmetric part}} + \underbrace{\tfrac{1}{2}(A - A^T)}_{\text{asymmetric part}} \tag{2}$$

thus, symmetrizing a matrix means neglecting its asymmetric part.

Following this consideration, the tracer symmetric connectome was defined as the matrix whose entries $\hat{t}_{ij}$ are defined as:

$$\hat{t}_{ij} = \frac{t_{ij} + t_{ji}}{2} \tag{3}$$

where t_{ij} represents the original tracer connection strength between area *i* and area *j*.

The symmetric tracer structural connectivity is shown in Figure 2A.

Asymmetrized dMRI connectome:

As opposed to symmetrizing a matrix which is a straightforward procedure, a-symmetrizing a matrix is an ill-posed problem, since it means introducing a new degree of freedom in the system, and not a unique solution exists. Thus, to find the asymmetric version of the dMRI connectome we assumed some constraints: we injected in each connection the same degree of asymmetry contained in the respective tracer connection, while preserving the dMRI weight balancing. In other words, our asymmetrization method assumes that the degree of asymmetry is independent on the connection strength value.

We defined the asymmetry degree μ_{ij} between connection *i* and connection *j* as:

$$\mu_{ij} = \begin{cases} \frac{t_{ij}}{t_{ji}}, \wedge\ t_{ij} \le t_{ji} \\ \frac{t_{ji}}{t_{ij}}, \wedge\ t_{ij} > t_{ji} \end{cases} \tag{4}$$

so that:

if the *ij* connection is symmetric: $t_{ij} = t_{ji} \Rightarrow \mu_{ij} = +1$

if the *ij* connection is anti-symmetric: $t_{ij} = -t_{ji} \Rightarrow \mu_{ij} = -1$

However, since the connection strengths in the connectome are always positively defined, μ_{ij} is a value always between 0 and 1.

The information on the directionality of the tracer connection between area *i* and area *j*, measured by μ_{ij}, are inserted in the diffusion-MRI connectome by modifying the original connection w_{ij} in $\check{w}_{ij}$:

$$\mu_{ij} = \frac{t_{ij}}{t_{ji}} = \frac{\check{w}_{ij}}{\check{w}_{ji}} \tag{5}$$

Specifically, we defined $\check{w}_{ij} = w_{ij} - k$ and $\check{w}_{ji} = w_{ji} + k$, where k is defined as:

$$\mu_{ij} = \frac{\check{w}_{ij}}{\check{w}_{ji}} = \frac{w_{ij}-k}{w_{ji}+k} \Rightarrow k = w_{ij}\frac{1-\mu_{ij}}{1+\mu_{ij}} \tag{6}$$

It is important to notice that the asymmetrization of the connectome does not imply the introduction of new connections: if the original diffusion-MRI connection w_{ij} is absent it follows, from the last equation, that also the increment k will be zero.

The asymmetrized deterministic connectome is shown in Figure 2B.

<u>Hybrid connectome: the role of individual connections</u>

We aimed to study the influence of the technique, the dMRI or the tracer one, in reconstructing the connections of a specific brain area. For this purpose, we built surrogate connectomes where all the brain wirings were reconstructed with deterministic dMRI except the connections of the region under examination that were measured with anatomical tracing.

In particular, for each deterministic dMRI connectome *W*, composed of N brain areas, we generated *N* different connectomes W^k by substituting the incoming and outgoing non-zero dMRI connections of area k with the corresponding tracer connections. The entry $w_{ij}^k$of the hybrid connectome W^k are defined as:

$$w_{ij}^k = \begin{cases} w_{ij} \text{if} i,j \in [1,2,\dots,k+1,k-1,\dots,N] \\ t_{kj} \text{if} i = k \text{and} w_{ij} \neq 0 \\ t_{ik} \text{if} j = k \text{and} w_{ij} \neq 0 \end{cases}$$

where w_{ij} and t_{ij} represent the connection strength of the original-individual deterministic dMRI and the original tracer connectome, respectively.

It is important to notice that this operation does not imply the introduction of new connections.

5. Comparing anatomical connectivities

U-static as a measure of connectome similarity

We used the Mann-Whitney test to check if the connections strength of connectomes W_i and W_j come from the same distribution. The null hypothesis of the test, H_0, is that the probability of an observation, i.e. a connection strength, of the connectome W_i exceeding an observation from population W_j equals the probability of an observation W_j exceeding an observation from sample W_i:

$$H_0: P(W_i > W_j) = P(W_i < W_j)$$

the alternative hypothesis, H_1, is:

$$H_1: P(W_i > W_j) \neq P(W_i < W_j)$$

The test involves the calculation of a statistic, usually called U.

For sample size above 20, which is our case, the distribution of the U variable under the null hypothesis can be approximated using the normal distribution. The U variable ranges between 0 and $n_1 n_2$, where n_1 and n_2 are the dimensionalities of the two connectomes. For $U \leq U^* = n_1 n_2/2$ the test states that the H_0 can be rejected.

It follows that it is possible to normalize the U value between 0 and 1, by dividing it by the product of the dimensionality of the two connectomes; in this case the discriminator value U^* is 0.5.

Euclidean distance as quantification of hemispheric functional lateralization:

We quantified the functional lateralization of a given region *x* as the Euclidean distance between the functional connections of the left area *x* and the functional connections of the right area *x*.

6. Simulated resting state dynamics

Using the previously described connectomes we conceptualized the mouse brain as a neuronal network. The mean activity of each brain region, i.e. the network's node, was defined by the reduced Wong Wang model (11). In this approach, the dynamics of a region is given by the whole dynamics of excitatory and inhibitory populations of leaky integrate-and-fire neurons interconnected via NMDA synapses. Here we take into account the model with a further reduction performed in (12): the dynamics of the output synaptic NMDA gating variable S of the *i*-th brain area is strictly bound to the collective firing rate H_i. The resulting model is given by the following coupled equations:

$$\frac{dS_i}{dt} = \frac{-S_i}{\tau_s} + (1 - S_i)\gamma H_i + \sigma\eta_i(t) \quad (7)$$

$$H_i = \frac{ax_i - b}{1 - exp\left(-d(ax_i - b)\right)} \quad (8)$$

$$x_i = \omega J_N S_i + J_N G \sum_j w_{ij} S_j + I_o \quad (9)$$

where x_i is the synaptic input to the i-th region. γ is a kinetic parameter fixed to 0.641, τ_s is the NMDA decay time constant and its value is 100 ms; a, b and d are the parameters of the input and output function H and are respectively equal to 270 nC^{-1}, 108 Hz, 0.154 s. $J_N = 0.2609nA$ is an intensity scale for the synaptic input current. ω is the local excitatory recurrence and I_o is the external input current. G is the coupling strength i.e. a scalar parameter which scales all the connection strengths w_{ij} without altering the global topology of the network. We set the noise amplitude σ of the normally distributed stochastic variable η_i to 0.015 since this level of noise is able to sustain brain states oscillations.

The external input current, I_o, and the local excitatory recurrence, ω, are set to 0.3 nA and 1, respectively, in order to enrich the non-linearity of the dynamics of each brain region. In this case, studying the dynamics of isolated brain areas ($G = 0$ in equation (9)), it is possible to notice that each brain area is in a bistable state and it oscillates between high and low activity fixed points (13). It has been noticed in (13) that enriching the non-linearity of each brain areas introduces global network's attractors that are not in trivial relation with the anatomical connectivity; this model offers the chance to reproduce the non-stationary features of the functional connectivity patterns, as shown by the checkboard pattern of the simulated FCD in Figure S2b.

For each connectome, we identified the coupling strength values for which the system is experiencing multistability. The optimal coupling strength range is defined as the values for which the system low and high states coexist, and it is identified by building the system's bifurcation diagram as described in (12).

The brain activity, for each connectome, is simulated for 40 values of coupling strength that equally span between 0 and M, where M corresponds to the coupling strength value for which the low state (identified with the previous method), disappears. The simulations obtained from each connectome, for different coupling strength value, are used to calculate the predictive power of the connectome as explained in the section.

Integration scheme and BOLD signals

Model equations are numerically solved using the Euler Maruyama integration method with a fixed integration step of 0.1 ms. Simulated BOLD signal is obtained by converting the simulated synaptic

activity (equation (7)) using the Balloon-Windkessel method (14) with the default value implemented in The Virtual Brain (15).
The BOLD time-series are down-sampled to 2.5 sec according to the temporal resolution of the experimental data.

7. Resting state signals analysis

Functional connections in the experimental and simulated time-series are explored from both spatial and temporal point of views using the Functional Connectivity (FC) and the Functional Connectivity Dynamics (FCD), respectively. We also explored the relation between functional links by estimating the Functional Meta-Connectivity (FMC).

Functional Connectivity (FC)

The FC matrix is defined as the matrix whose *ij*-th element is the Pearson correlation between the BOLD signal of the brain region *i* and of the brain region *j*. An example of empirical and simulated FC is shown in Figure S1.

Functional Connectivity Dynamics (FCD)

The FCD matrix for the experimental and simulated signals is calculated using the sliding windows approach (13, 16).
To estimate the FCD, the entire BOLD time-series is divided in time windows of a fixed length (2 min) and with a spanning of 2.5 sec; the data points within each window centered at the time $t_i$were used to calculate FC(t_i).
The *ij*-th element of the FCD matrix is calculated as the Pearson correlation between the upper triangular part of the FC(t_i) matrix arranged as a vector and the upper triangular part of the FC(t_j) matrix arranged as a vector. In order to observe signal correlations at frequency greater than the typical one of the BOLD signals, the sliding window length is fixed to 2 min, since, as demonstrated by (17), the non-spurious correlations in the FCD are limited by high-pass filtering of the signals with a cut-off equal to the inverse of the window length.
An example of empirical and simulated FCD is shown in Figure S2.
The typical FCD matrix during resting-state has a checkboard appearance (see experimental FCD in Figure S2) indicating that the system is switching between stable networks configuration (13, 16). We quantified the *switching degree* of the simulated and experimental system as the variance of the triangular part of the FCD once excluded the overlapping entries (i.e. the entries of the FCD

matrix that quantify the correlation of FCs calculated over the sliding window of overlapping time interval). We called this quantity clue of switching (cs).

Functional Meta-Connectivity (FMC)

To compare the dynamical evolution of the functional connections between different systems we calculate, for each system, the FMC. The FMC, of a BOLD signals of *N* areas, is a N^2xN^2 matrix that quantifies the inter-region functional correlation of the system. The *ij*-th element of the FMC represents the Pearson correlation between the temporal evolution of the *i*-th functional link and the *j*-th functional link.

8. Comparing experimental and simulated BOLD signals

We quantified the ability of a given connectome to be used as a skeleton of the virtual system by comparing the accordance between the simulated functional connections, generated using that connectome, and the functional connections experimentally recorded in the resting state sessions. As discussed before, we used the FC as the metric for quantifying the experimental and simulated functional connections. Indeed, although the FC metric is not able to capture the non-stationary nature of the resting state signals, the static functional connections are stable across resting state recordings in the same animal; on the other hand, FMC, that is able to quantify the dynamical evolution of the functional connections, is not sufficiently stable across resting state recordings (see Figure S2), and thus cannot be used for quantifying the goodness of the simulated activity.

We use 120 experimental resting state sessions recorded in 19 different animals, thus 120 experimental FCs (eFCs). For each experimental resting state session, *d*, recorded in mouse, *m,* we built a corresponding virtual mouse brain able to mimic resting state activity:

A. In the case of not-individual connectomes (i.e. the Allen SC, surrogate and original ones, and the averaged dMRI connectome) for each recording session, irrespective to the mouse scanned, we build a virtual mouse brain using the same connectome. It follows that we have 120 virtual mouse brains that are identical from the structural network point of view; however it is important to remark that virtual mouse brain with identical anatomical structure are not necessarily identical from a dynamical point of view since changing the parameters of the model one can obtain different resting state dynamics. It is this variety in dynamics that allows us to model 120 different resting state sessions using the same anatomical structure.
B. Instead, in the case of personal dMRI connectomes, surrogate and original ones, we used the information of mouse *m* to simulate the corresponding experimental resting state session. It

follows that we have 120 virtual mouse brains defined according to 19 different connectomes, and thus, differently from case A, here virtual mouse brains are different both from the anatomical and dynamical point of view.

In these virtual brains, we simulate resting state activity using the reduced Wong Wang model in the bistable configuration. In the model there is a parameter, i.e. the coupling strength G (eq. 9), whose value is not fixed by biological constraints, but is variable and is used to optimize the simulated output (13, 18, 19). For each recording session, thus for each eFC, we optimize the coupling strength value in order to maximize the correlation between the eFC and the simulated FC (sFC). In particular, for each connectome, we explored 40 different values of the coupling strength in an equally spanned interval range. The interval range is identified according to the bifurcation diagram of each connectome built as described in (12).

For each mouse, *m*, and each session, *d*, we defined the PP of a given connectome c as the maximum Pearson correlation between the empirical FC (eFC) and the simulated FC (sFC) obtained for the different coupling strength values G:

$$PP(c,m,d) = \max_{G}\{corr[sFC(c,G), eFC(m,d)]\}$$

The PP of a given connectome $PP(c)$ is the mean over all the mice and the sessions of the $PP(c,m,d)$:

$$PP(c) = \underset{m,d}{mean}\{PP(c,m,d)\}$$

Since 120 experimental resting state sessions enter in the analysis, it follows that for each type of connectome *c* the average PP is calculated over 120 PP values. In order to assess the significance of the difference in PP of differently derived connectomes we used the p-value calculated through the Welch's test; we corrected the p-values for multiple comparisons using the Bonferroni correction. We measure the effect size using the Cohen's d and we calculated the 95% confidence intervals (CIs) using the estimation stats framework as described in (20) and available at https://www.estimationstats.com/.

Finally, we want to point out that the simulated functional network is composed of more areas than the experimental one since the simulation is based on the anatomical information that has a greater spatial resolution than the functional one. Thus, in order to correlate the eFC and the sFC we reduced them to the same number of areas.

9. Data sharing

All imaging raw data and the relevant codes used in this study are available in BIDS format on OpenNeuro, https://openneuro.org/datasets/ds002307.

REFERENCES

1. Bergmann E, Zur G, Bershadsky G, Kahn I (2016) The organization of mouse and human cortico-hippocampal networks estimated by intrinsic functional connectivity. *Cereb Cortex*:1–16.
2. H. Manouze *et al.*, Effects of single cage housing on stress, cognitive, and seizure parameters in the rat and mouse pilocarpine models of epilepsy. *eNeuro* **6**, ENEURO.0179-18.2019 (22 July 2019).
3. Oh SW, et al. (2014) A mesoscale connectome of the mouse brain. *Nature* 508(7495):207.
4. Sanz Leon P, et al. (2013) The Virtual Brain: a simulator of primate brain network dynamics. *Front Neuroinformatics* 7:10.
5. Melozzi F, Woodman MM, Jirsa VK, Bernard C (2017) The Virtual Mouse Brain: A Computational Neuroinformatics Platform To Study Whole Mouse Brain Dynamics. *eNeuro*:ENEURO–0111.
6. Greve DN, Fischl B (2009) Accurate and robust brain image alignment using boundary-based registration. *Neuroimage* 48(1):63–72.
7. Tournier J-D, Calamante F, Connelly A (2012) MRtrix: diffusion tractography in crossing fiber regions. *Int J Imaging Syst Technol* 22(1):53–66.
8. Tournier J-D, Calamante F, Connelly A (2007) Robust determination of the fibre orientation distribution in diffusion MRI: non-negativity constrained super-resolved spherical deconvolution. *Neuroimage* 35(4):1459–1472.
9. Tournier JD, Calamante F, Connelly A (2010) Improved probabilistic streamlines tractography by 2nd order integration over fibre orientation distributions. *Proceedings of the International Society for Magnetic Resonance in Medicine*, p 1670.
10. Smith RE, Tournier J-D, Calamante F, Connelly A (2013) SIFT: spherical-deconvolution informed filtering of tractograms. *Neuroimage* 67:298–312.
11. Wong K-F, Wang X-J (2006) A recurrent network mechanism of time integration in perceptual decisions. *J Neurosci* 26(4):1314–1328.
12. Deco G, et al. (2013) Resting-state functional connectivity emerges from structurally and dynamically shaped slow linear fluctuations. *J Neurosci* 33(27):11239–11252.
13. Hansen EC, Battaglia D, Spiegler A, Deco G, Jirsa VK (2015) Functional connectivity dynamics: modeling the switching behavior of the resting state. *Neuroimage* 105:525–535.
14. Friston KJ, Mechelli A, Turner R, Price CJ (2000) Nonlinear responses in fMRI: the Balloon model, Volterra kernels, and other hemodynamics. *NeuroImage* 12(4):466–477.
15. Sanz-Leon P, Knock SA, Spiegler A, Jirsa VK (2015) Mathematical framework for large-scale brain network modeling in The Virtual Brain. *Neuroimage* 111:385–430.
16. Allen EA, et al. (2014) Tracking whole-brain connectivity dynamics in the resting state. *Cereb Cortex* 24(3):663–676.
17. Leonardi N, Van De Ville D (2015) On spurious and real fluctuations of dynamic functional connectivity during rest. *Neuroimage* 104:430–436.
18. Deco G, Jirsa VK, McIntosh AR (2011) Emerging concepts for the dynamical organization of resting-state activity in the brain. *Nat Rev Neurosci* 12(1):43.
19. Deco G, Jirsa VK, McIntosh AR (2013) Resting brains never rest: computational insights into potential cognitive architectures. *Trends Neurosci* 36(5):268–274.
20. Ho J, Tumkaya T, Aryal S, Choi H, Claridge-Chang A (2019) Moving beyond P values: data analysis with estimation graphics. *Nat Methods*:1.

Table S1: List of the acronyms for the different brain regions considered in this work.

Acronym	Complete name
ACB	Nucleus accumbens
ACAd	Anterior cingulate area, dorsal part
ACAv	Anterior cingulate area, ventral part
AId	Agranular insular area, dorsal part
AN	Ansiform lobule
AON	Anterior olfactory nucleus
AUDd	Dorsal auditory
AUDp	Primary auditory area
AUDv	Ventral auditory area
CA1	Field CA1
CA3	Field CA3
CENT	Central lobule
COPY	Copula pyramidis
CP	Caudoputamen
CUL	Culmen
DG	Dentate gyrus
ECT	Ectorhinal area
ENTl	Entorhinal area, lateral part
ENTm	Entorhinal area, medial part, dorsal zone
GRN	Gigantocellular reticular nucleus
GU	Gustatory areas
IC	Inferior colliculus
IRN	Intermediate reticular nucleus
LHA	Lateral hypothalamic area
LSr	Lateral septal nucleus, rostral (rostroventral) part
MOB	Main olfactory bulb
MOp	Primary motor area
MOs	Secondary motor area
MRN	Midbrain reticular nucleus
OT	Olfactory tubercle
PAG	Periaqueductal gray
PERI	Perirhinal area

PFL	Paraflocculus
PIR	Piriform area
PRM	Paramedian lobule
PRNc	Pontine reticular nucleus, caudal part
PRNr	Pontine reticular nucleus
RSPd	Retrosplenial area, dorsal part
RSPv	Retrosplenial area, ventral part
SCm	Superior colliculus, motor related
SCs	Superior colliculus, sensory related
SI	Substantia innominata
SIM	Simple lobule
SSp-bfd	Primary somatosensory area, barrel field
SSp-m	Primary somatosensory area, mouth
SSp-n	Primary somatosensory area, nose
SSp-ul	Primary somatosensory area, upper limb
SSs	Supplemental somatosensory area
SUB	Subiculum
TEa	Temporal association areas
UVU	Uvula (IX)
VISp	Primary visual area

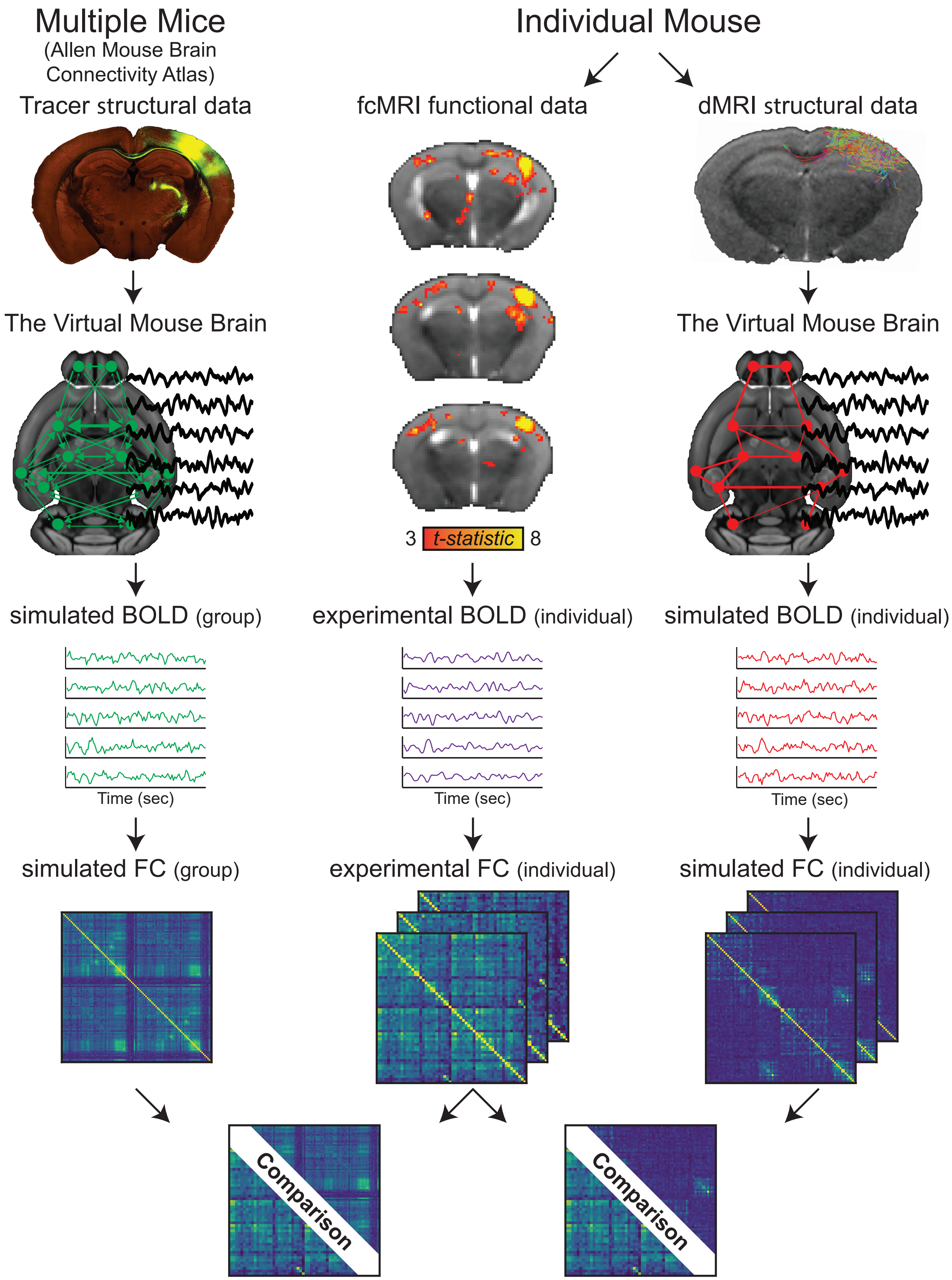
Multiple Mice
(Allen Mouse Brain
Connectivity Atlas)
Tracer structural data
Individual Mouse
fcMRI functional data
dMRI structural data
The Virtual Mouse Brain
The Virtual Mouse Brain
3 t-statistic 8
simulated BOLD (group)
experimental BOLD (individual)
simulated BOLD (individual)
Time (sec)
Time (sec)
Time (sec)
simulated FC (group)
experimental FC (individual)
simulated FC (individual)
Comparison
Comparison

Figure S1:

Figure S1: Experimental workflow. Each mouse is scanned to obtain both SC and FC data. First, FC is estimated empirically for each subject as illustrated by the seed-based analysis of the barrel-related primary somatosensory cortex (t-test of 7 recording sessions of the same mouse, $p < 0.01$, uncorrected, voxel extent = 20) and FC matrices. Then, simulated BOLD activity is generated by the virtual mouse brain using dMRI-based SC. The simulated and experimental brain dynamics are compared through a static-FC metric to estimate the predictive power. Then, the results are compared to the predictive power of the gold standard Allen SC. Note that the Allen SC, but not dMRI-based SC, provides fiber directionality.

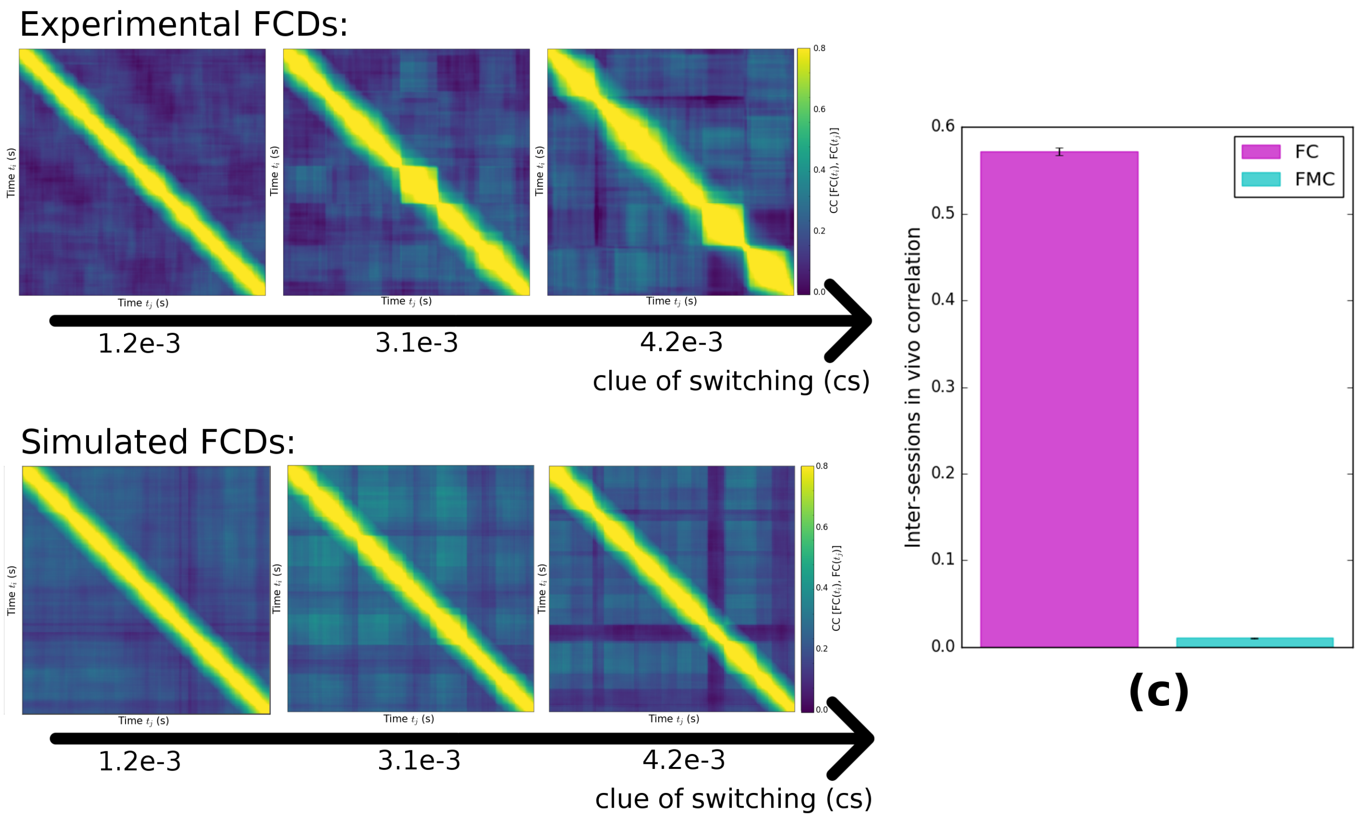

Figure S2: Variability of FCD metric in experimental (a) and simulated (b) data. FCDs on the top are calculated from the experimental resting state data from 3 different scanning sessions; FCDs on the bottom are calculated from simulated resting state data; in both case we use a sliding window length of 2 minutes and a spanning of 2.5 seconds. We quantify the presence of the switching, i.e. the checkboard pattern in the FCD matrix, as the variance of the triangular part of the FCD, once excluded the overlapping entries. We call this quantity: clue of switching (cs). The cs value is indicated below each FCD. FCDs are ordered for increasing cs values. The checkboard pattern appears more clearly as cs increases.

(c) The height of the bar represents the Pearson correlation between inter-sessions for experimental FC (magenta bar) and FMC (blue bar). The result shows that the FMC matrix can not be considered as a metric since FMC is poorly reproducible across sessions in the same animal.

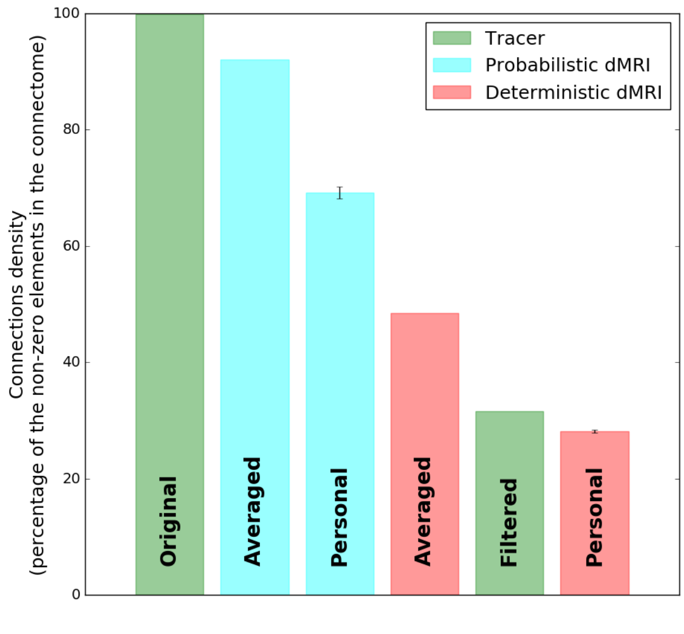

(a)

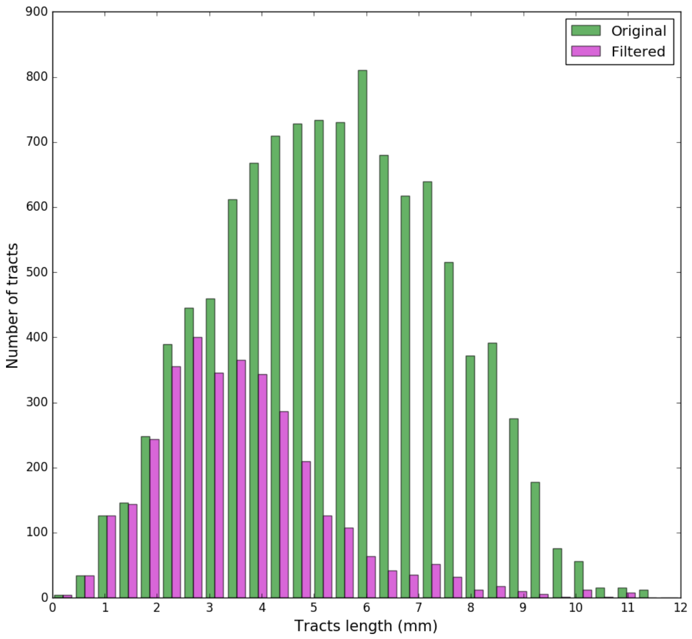

(b)

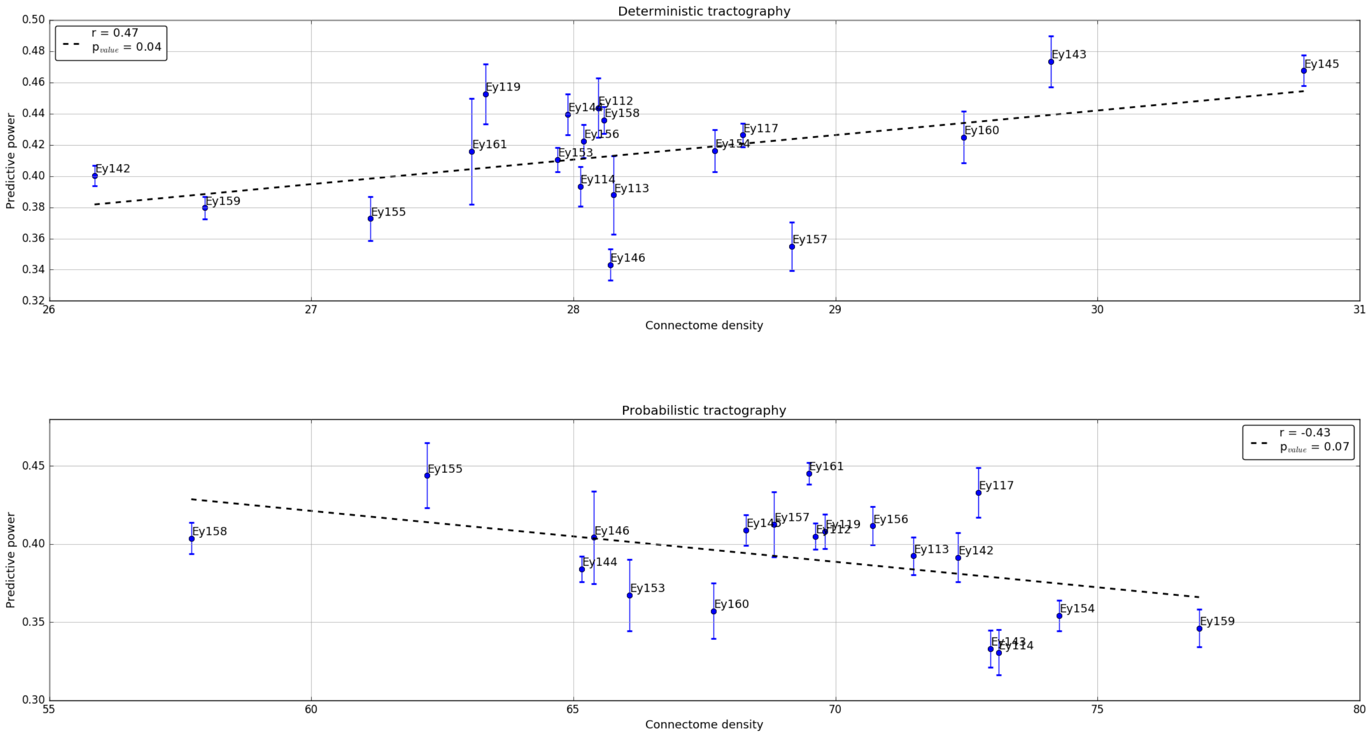

(c)

Figure S3

Figure S3: (a) The height of the bars represents the density of the connectome used in this study. The difference in the rank of the bars in this plot and those representing the connectomes PP (Figure 2-3) highlights the absence of a relation between the connectome's density and its PP. In line with this observation, the plots in (c) show the relation between the PP and the density of the dMRI-based connectomes processed with the deterministic tractography (plot on the top) and probabilistic tractography (plot in the bottom).The relation between the connectomes density and the PP it is opposite between the case of deterministic and probabilistic processing. In the case of deterministic processed tractography data, denser connectomes, i.e. with less false negative, have a greater PP than sparser connectomes, that is connectomes with more false negative. Conversely, in the case of probabilistic processed data, sparser connectomes, i.e. with less false negative, have a greater PP than denser connectome. From the results of the panels (a) and (c) it follows that the number of connections in a connectome does not directly relate with its ability in predicting brain dynamics.

The histogram in (b) shows the distribution of the fibers lengths included in the original tracer-based (green bars) and in the filtered tracer-based (magenta bars) connectome. The filtered connectome was obtained from the original tracer connectome by removing the connections not present in at least one of the 19 deterministic connectomes (68% of the tracer-based connections removed). This operation results in removing mainly long-range connections (the mean length of the tracts contained in the original tracer connectome and in the filtered one is respectively 5.40±0.02 mm and 3.57±0.03 mm).

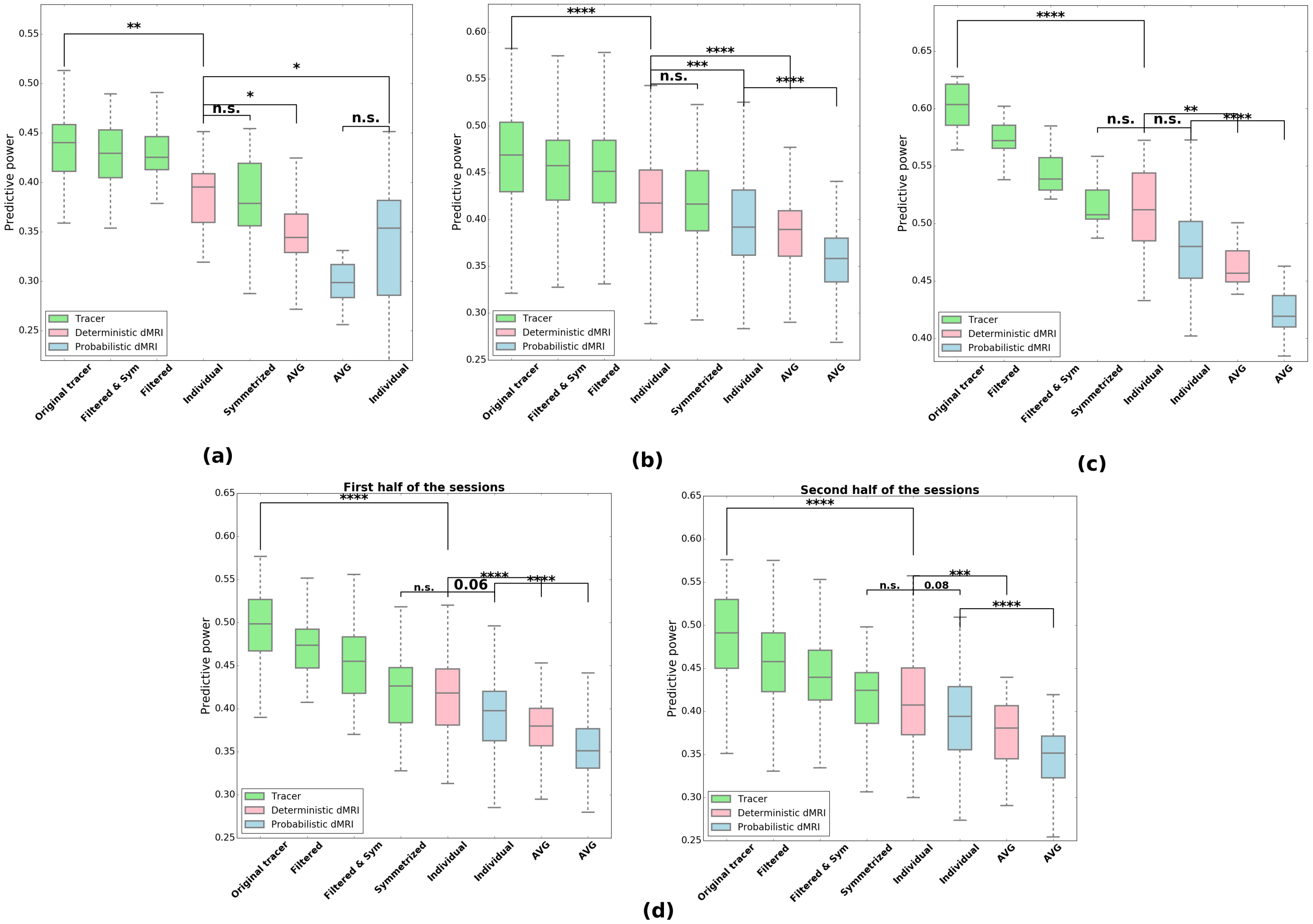

Figure S4: The box plots represent the predictive power of the tracer-based (green bars), deterministic dMRI-based (red bars) and probabilistic dMRI-based (blue bars) connectomes. The meaning and the construction of the figures are analogous to Figure 3b 3C in the main text.

(a) Results obtained for examination of 7 wild type inbred C57BL/6 mice (4±2 sessions per animal, 11±4 minutes per session, mean±SD). The results are similar to those found for in hybrid mice.

(b) Results for hybrid mice obtained using experimental resting state data preprocessed using global signal regression. The results are consistent to those presented in Figure 2 and Figure 3, that are those obtained from experimental resting state data not not globally signals regressed.

(c) Results obtained after averaging recording sessions in the same animal. The predictive power trend is analogous to the one obtained considering separately the contribution of each recording session.

(d) The two box plots show the predictive power calculated splitting the recoding sessions in two; results are consistent with the one obtained considering the complete dataset.

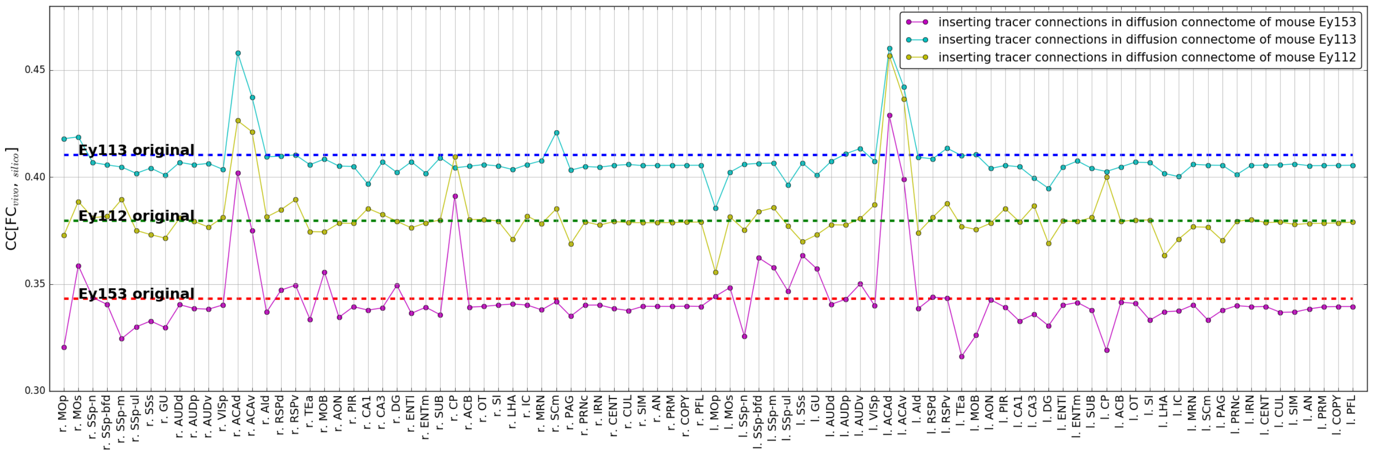

Figure S5: Dots of different colors represent the predictive power of the deterministic dMRI connectome of different mice (namely Ey113, Ey112 and Ey153) with the connections of the brain area (x-axis) replaced with the corresponding tracer connections. Red, green and blue dashed lines represent the predictive power of the personal deterministic dMRI connectome of different mice. The figure shows that the change in predictive power strictly depends on the considered brain areas and on the mouse connectome: for example, the replacement of the right caudoputamen connections strongly enhances the predictive power of the deterministic dMRI connectome of mice Ey153 and Ey112, but not of mouse Ey113. Conversely, the replacement of cerebellum's connections does not really affect the performance of the connectome in predicting brain dynamics. The labels of brain areas are shown in table S1.

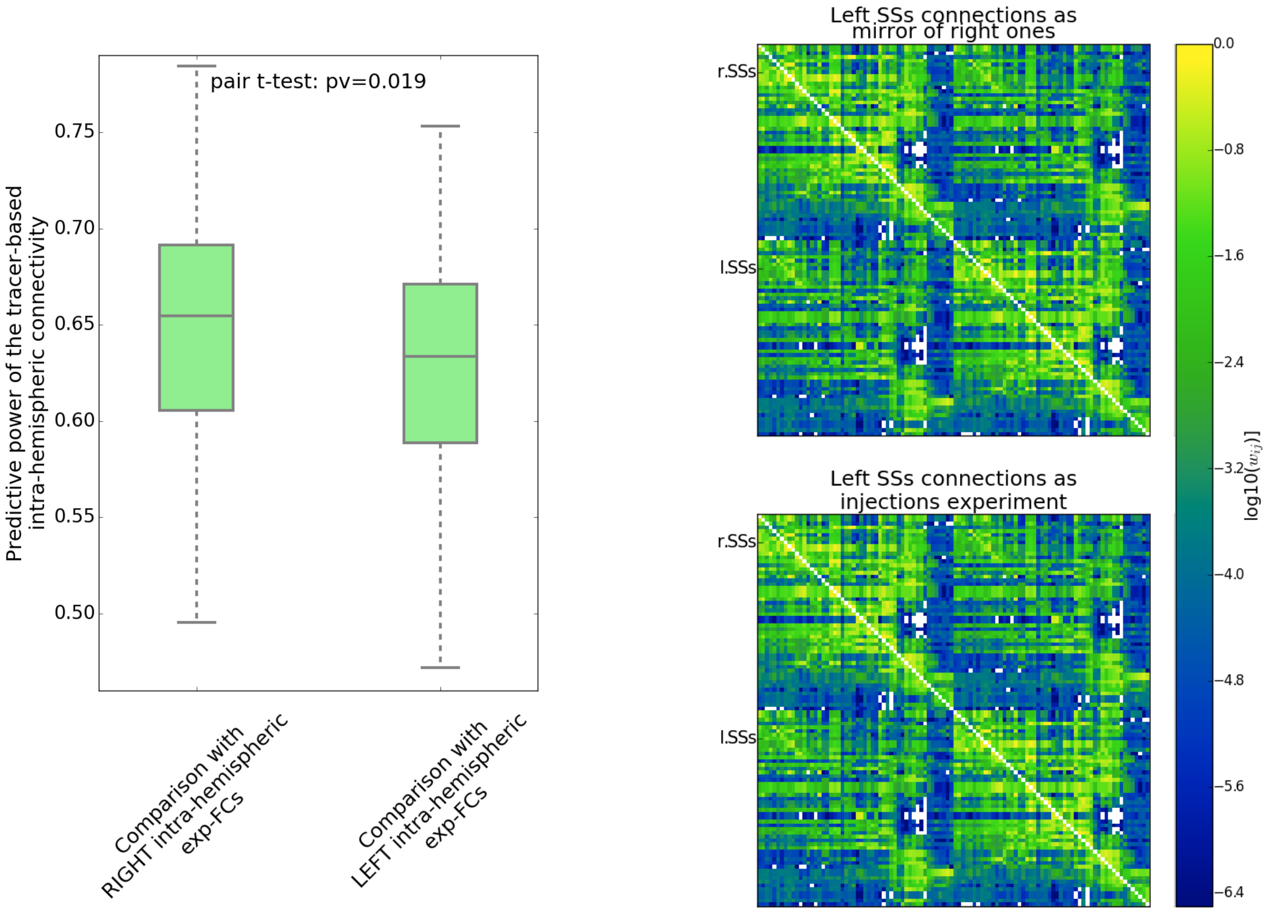

(a) (b)

Figure S6: (a)The box plot represent the predictive power of the tracer-based connectome built using only intra-hemispheric connections. The predictive power is estimated comparing the predictions of the connectome with the intra-hemispheric experimental functional connections measured from the right (left bar) or the left (right bar) hemisphere. The difference in the predictive power shows that intra-hemispheric tracer-based connections, obtained after injecting the compound in the right hemisphere, are able to predict better right than left intra-hemispheric functional connections. The result suggests that the mouse brain is lateralized.
(b) Tracer-based connectomes built using the information of one injection experiment per area. The difference between the two connectomes relies on the definition of the connections of the left SSs: in the connectome on the top, the connections of the left SSs are the mirror image of the connections of the right SSs, that are connections built using information from an injection experiment performed in the right SSs region. In contrast, in the connectome shown on the bottom, the left SSs connections are built using the information of an injection experiment performed in the left SSs area.
